# “Jack‐of‐all‐trades” is parthenogenetic

**DOI:** 10.1002/ece3.9036

**Published:** 2022-06-23

**Authors:** Mark Maraun, Paul S. P. Bischof, Finn L. Klemp, Jule Pollack, Linnea Raab, Jan Schmerbach, Ina Schaefer, Stefan Scheu, Tancredi Caruso

**Affiliations:** ^1^ JFB Institute of Zoology and Anthropology Georg August University Göttingen Göttingen Germany; ^2^ School of Biology and Environmental Science University College Dublin Dublin Ireland

**Keywords:** frozen niche variation, general‐purpose genotype, generalism, oribatid mites, parthenogenesis, range size, sex, specialism

## Abstract

Sex is evolutionarily more costly than parthenogenesis, evolutionary ecologists therefore wonder why sex is much more frequent than parthenogenesis in the majority of animal lineages. Intriguingly, parthenogenetic individuals and species are as common as or even more common than sexuals in some major and putative ancient animal lineages such as oribatid mites and rotifers. Here, we analyzed oribatid mites (Acari: Oribatida) as a model group because these mites are ancient (early Paleozoic), widely distributed around the globe, and include a high number of parthenogenetic species, which often co‐exist with sexual oribatid mite species. There is evidence that the reproductive mode is phylogenetically conserved in oribatid mites, which makes them an ideal model to test hypotheses on the relationship between reproductive mode and species' ecological strategies. We used oribatid mites to test the frozen niche variation hypothesis; we hypothesized that parthenogenetic oribatid mites occupy narrow specialized ecological niches. We used the geographic range of species as a proxy for specialization as specialized species typically do have narrower geographic ranges than generalistic species. After correcting for phylogenetic signal in reproductive mode and demonstrating that geographic range size has no phylogenetic signal, we found that parthenogenetic lineages have a higher probability to have broader geographic ranges than sexual species arguing against the frozen niche variation hypothesis. Rather, the results suggest that parthenogenetic oribatid mite species are more generalistic than sexual species supporting the general‐purpose genotype hypothesis. The reason why parthenogenetic oribatid mite species are generalists with wide geographic range sizes might be that they are of ancient origin reflecting that they adapted to varying environmental conditions during evolutionary history. Overall, our findings indicate that parthenogenetic oribatid mite species possess a widely adapted general‐purpose genotype and therefore might be viewed as “Jack‐of‐all‐trades.”

## Graphical abstract

Our results suggest that parthenogenetic oribatid mite species are more generalistic than sexual species supporting the general purpose genotype hypothesis. Our findings indicate that parthenogenetic oribatid mite species possess a widely adapted general‐purpose genotype and therefore might be viewed as “Jack‐of‐all‐trades”.

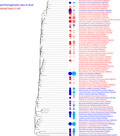

## 1 INTRODUCTION

Sexual reproduction dominates in nearly all plant and animal taxa despite being associated with breaking up favorable gene combinations and the production of males producing no offspring themselves (Bell, 1982; Lehtonen et al., 2012; Maynard Smith, 1971, 1978; Otto, 2009). Understanding the reasons for the dominance of sexual reproduction over parthenogenesis posed a major challenge for evolutionary biology for long (Burke & Bonduriansky, 2017; Lively, 2010; Morran et al., 2011; Scheu & Drossel, 2007; Song et al., 2011), and no general conclusions have yet emerged (Neiman et al., 2018; West et al., 1999). We propose that investigating animal lineages in which parthenogenesis is common can help shedding light on this conundrum. This is particularly true when analyzing taxa that have maintained parthenogenesis as the main reproductive mode for tens of millions of years (Neiman & Schwander, 2011). This applies to bdelloid rotifers (Ricci, 2017), darwinulid ostracods (Schön et al., 2012), and several clusters of species of oribatid mites (Pachl et al., 2021). Understanding their long‐term persistence received increased attention in the last decades as it may contribute to a better understanding of the dominance of sexual reproduction in the animal kingdom (Neiman & Schwander, 2011). All of these taxa have survived over long evolutionary periods of time without sexual reproduction (Butlin, 2002; Mark Welch & Meselson, 2000; Martens et al., 2003), although this also has been disputed (Schwander, 2016). In fact, bdelloid rotifers may engage in some form of noncanonical sex (Signorovitch et al., 2015) and rare males have been observed in darwinulid ostracods (Smith et al., 2006) and parthenogenetic oribatid mites (Norton & Palmer, 1991).

In oribatid mites, sexual and parthenogenetic lineages co‐occur, which provides the opportunity to test hypotheses on the relationship between parthenogenesis, sexual reproduction, phylogeny, and ecology over evolutionary periods of time. In oribatid mites, there is no evidence for intraspecific variation of the reproductive mode. Additionally, no case of geographic parthenogenesis is known. This has not been investigated for all species of oribatid mites, but since there are several studies that have investigated sex ratios of oribatid mite species (see Table S2 for details), variation in the reproductive mode within species is unlikely to have not gone unnoticed. Oribatid mites are mainly living in litter and soil, but some species are arboreal and few are aquatic (Norton & Behan‐Pelletier, 2009). They are mainly decomposers and fungal feeders, but some species feed on algae, mosses, or lichens or are even predatory (Maraun et al., 2011). Oribatid mites likely originated in Cambrian or Precambrian times (Arribas et al., 2019; Schaefer et al., 2010), and several lineages comprising clusters of parthenogenetic species are of ancient origin (Pachl et al., 2021). Parthenogenetic oribatid mite lineages likely radiated into species‐rich clusters of morphologically distinct species, which is an enigma on its own (Maraun et al., 2004), and their survival for tens of millions of years contradicts the commonly held view that parthenogenetic lineages are doomed to extinction due to the accumulation of deleterious mutations (Butlin, 2002; Maynard Smith, 1971).

Two hypotheses have been proposed to explain the occurrence and survival of parthenogenetic lineages. The frozen niche variation (FNV) hypothesis posits that parthenogenetic species comprise a number of well‐adapted specialized lineages with each of them possessing a different “frozen” genotype that all‐in‐all occupies a range of narrow niches and can even displace sexual taxa being better adapted to the available niches than their (comparatively generalistic) sexual progenitors (Vrijenhoek, 1979, 1984; Vrijenhoek & Parker, 2009). Although each parthenogenetic lineage might occupy a narrow ecological niche the sum of all these niches may be broader than in competing sexual species. These parthenogenetic lineages are usually assumed to be relatively young (Hörandl, 2009; Johnson & Bragg, 1999; Schön et al., 2000; Strasburg & Kearney, 2005). By contrast, the general‐purpose genotype (GPG) hypothesis posits that parthenogenetic lineages have broadly adapted genotypes that tolerate a wide range of environmental conditions (Baker, 1965; Lynch, 1984). Generalist species are assumed to evolve in heterogeneous habitats because specialized genotypes vanish in the long term due to habitat heterogeneity in space and time. GPG species therefore likely are older than FNV genotypes. The two hypotheses have been debated, but no consensus has been reached and, depending on circumstances, either of them may apply (Bierzychudek, 1989; Browne & Wanigasekera, 2000; Kenny, 1996; Parker & Niklasson, 1995; Semlitsch et al., 1997; Van Doninck et al., 2002; Vorburger et al., 2003; Weider, 1993).

In this study, we evaluated the validity of the FNV and GPG theory for explaining the frequency of parthenogenetic reproduction in oribatid mites. Assuming that generalist species typically occupy wider ranges than specialist species (Alonso‐Marcos et al., 2019; Coughlan et al., 2017; Gaston, 2003; Hörandl, 2009; Kearney, 2005), we studied the range size, i.e., the currently known geographic distribution, of sexual and parthenogenetic oribatid mite species using the list of oribatid mite species of the world and their distribution range (Subías, 2004, 2021). As a null hypothesis, we postulated that there is no relationship between the range size of species and reproductive mode. To test this hypothesis, we assembled data on oribatid species' range sizes and reproductive modes, and controlled for phylogenetic signal in both range size and reproductive mode, with the latter known to be phylogenetically conserved in oribatid mites (Norton et al., 1993). Considering the old age of parthenogenetic oribatid mite lineages we expected the null hypothesis to be wrong and the range size of parthenogenetic species on average to be larger than that of sexual species conform to the GPG theory.

## 2 MATERIALS AND METHODS

### 2.1 Data collection

Data on the range size of oribatid mites were assembled from Subías (2004, 2021; http://bba.bioucm.es/cont/docs/RO_1.pdf). The size of geographic regions, such as Holarctic, Palaearctic, Subtropical, and Neotropical, was taken from Hawkins and Porter (2001), and from internet sources (for details see Table S1).

We included 656 species of the ca. 11.000 described oribatid mite species of which 475 reproduce sexually and 181 by parthenogenesis (Table S2). The reproductive mode was taken from the literature (Cianciolo & Norton, 2006; Domes et al., 2007; Fischer et al., 2010; Maraun et al., 2019; Norton et al., 1993; Norton & Palmer, 1991; Wehner et al., 2014) or inferred from the reproductive mode of closely related species. Species were selected from Subías (2004, 2021) and overlapped in large with the species used in the study of Maraun et al. (2019), which included all major lineages of oribatid mites.

### 2.2 Statistical analysis

For a first descriptive analysis, the range size of sexual and parthenogenetic species of oribatid mites was compared using the Wilcoxon test since data were not normally distributed and variances were not homogenous (Kolmogorov–Smirnov test and Levene test; both *p* < .05). Data are reported as means including data distribution in a violin plot. Statistical analyses were carried out using R (R Core Team, 2021).

For testing our null hypothesis we controlled for phylogenetic relatedness between taxa (Gotelli & Ellison, 2004; Kembel et al., 2010; Swendsen, 2020). For phylogeny‐based analyses that tested for the independence of traits (i.e., range size and reproductive mode) we constructed a phylogenetic tree at the family level, based on all 18S rDNA sequences available at GenBank (ncbi.nlm.nih.gov) to generate the most inclusive dataset possible. At lower taxonomic levels, such as genus or species, Genbank provided only a fraction of taxa, and using only these would have resulted in a considerably reduced dataset. Therefore, we downloaded as many 18S rDNA sequences as possible for each oribatid mite family to obtain the most inclusive dataset including sequences of 61 of the total of 95 families (Table S3).

The distribution range of oribatid mites may be species‐specific. However, we assume that the distribution ranges of closely related species can be used as a proxy for the distribution range of another species from the same family. We admit that this is a weakness of the study, but we are simply unable to fix it as it would imply the sequencing of all the missing species. Sequences were aligned with Muscle in AliView v1.27 (Larsson, 2014) using default settings and a Maximum Likelihood tree was built with IQ‐Tree Web Server (http://iqtree.cibiv.univie.ac.at). For further statistical analyses, the phylogenetic tree was pruned to contain only one taxon per family using the drop. tip function in R, only the earliest derived taxon of a monophyletic family was kept in the tree. If families were not monophyletic, one representative per clade was kept in the phylogeny. If families included species with different reproductive modes, we kept one parthenogenetic and one sexual species in the phylogeny. To test for phylogenetic signal in the range size of species, we used the mean of all range sizes of species within families to reduce variation in distribution ranges, because a number of species in families were unevenly distributed and ranged between 1 and 69. Then, we compared the distribution of range sizes of the reduced dataset between sexual and parthenogenetic species using ANOVA (without phylogenetic correction) and the R function phylANOVA (with phylogenetic correction based on the reduced dataset at the family level) following Garland et al. (1993), Harmon et al. (2008) and Revell (2012).

Further, we tested – using the reduced dataset – if differences in range size between reproductive modes are due to a phylogenetic signal using Pagel's lambda (Pagel, 1999) and Blomberg's K (Blomberg et al., 2003), and analyzed standardized contrast variance (PIC) with 1000 randomizations using the tip. shuffle function. The analyses were implemented using the R packages ape 5.5 (Paradis & Schliep, 2019), phylobase 0.8.1 (Hackathon et al., 2020), picante 1.8.2 (Kembel et al., 2010), phytools 0.7.8 (Revell, 2012), and geiger 2.0.7 (Harmon et al., 2008; R Core Team, 2021). Additionally, we tested for phylogenetic signal in reproductive mode using the phylo.d function implemented in R package caper (Fritz & Purvis, 2010; Orme et al., 2018).

Further, we conducted a phylogenetically corrected logistic regression for a final test of our null hypothesis. We used the geographic range of species as a predictor of the probability that a species is parthenogenetic. We fitted the logistic regression using the R function binaryPGLMM (package “ape”), which is based on Ives and Helmus (2011) and Ives and Garland Jr. (2014). This strategy was assumed to be appropriate because (as shown in the results) range size displayed no phylogenetic signal, whereas reproductive mode tended to be a conserved trait, and the phylogenetic tree allowed to formulate a phylogenetic covariance matrix in the response variable of the model.

## 3 RESULTS

### 3.1 Range size

**FIGURE 1:**
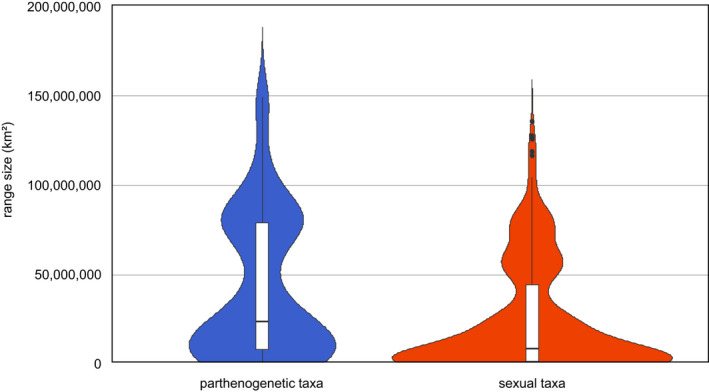
Truncated violin plot of geographic range sizes of parthenogenetic and sexual oribatid mite species including the median and the interquartile ranges. Whiskers show 95% confidence intervals (for statistical analysis, see text)

The range size of parthenogenetic taxa was significantly larger than that of sexual taxa (Wilcoxon rank test; *p* < .0001; Figure 1). On average, the range size of parthenogenetic species was 43,238,392 ± 41,417,072 km^2^ and that of sexual species 23,332,460 ± 30,005,194 km^2^.

### 3.2 Phylogeny

**FIGURE 2:**
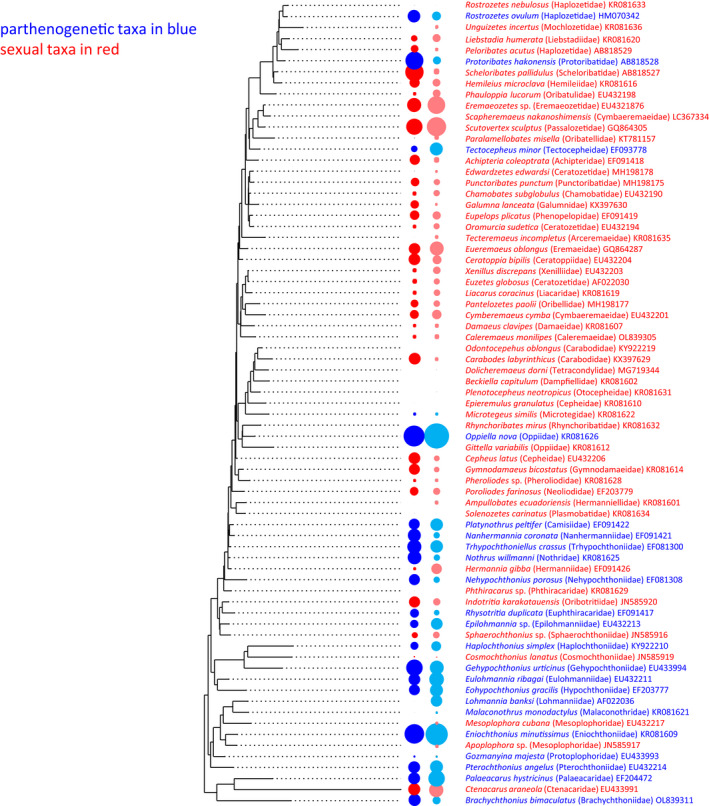
Maximum likelihood tree used in this study to test for phylogenetic constraints in geographic range sizes. Parthenogenetic species are indicated in blue, sexual species in red. The dark blue circles (left column) indicate the range size of the parthenogenetic species; the light blue circles (right column) indicate the mean range size of the parthenogenetic families; the dark red circles (left column) indicate the range size of the sexual species; the light red circles (right column) indicate the mean range size of the sexual families (very small range sizes are not visible)

The phylogenetic tree comprised 134 oribatid mite species and 8 outgroup taxa (3 Opilioacaridae, 3 Parasitiformes, 2 Trombidiformes; Table 3). Since the phylogeny of the Acari is not well resolved, and since it is not known if Acari are monophyletic, we decided to include a wide range of potential outgroup taxa. The reduced tree that represented one taxon per monophyletic family included 74 taxa (Figure 2). The phylANOVA was not significant (*F* = 8.07, *p* = .329), indicating that the difference in the geographic range of sexual and parthenogenetic taxa cannot be explained by phylogeny. The mean of all areas was within the normal distribution of 1000 randomized analyses. Pagel's lambda and Blomberg's K were not significant (lambda = 0.31, *p* = .31; K = 0.155, *p* = .21). The reproductive mode was phylogenetically conserved; it did not deviate significantly from the Brownian motion model (estimated D = −0.013, probability of E[D] resulting from Brownian phylogenetic structure = 0.53).

### 3.3 The model

**FIGURE 3:**
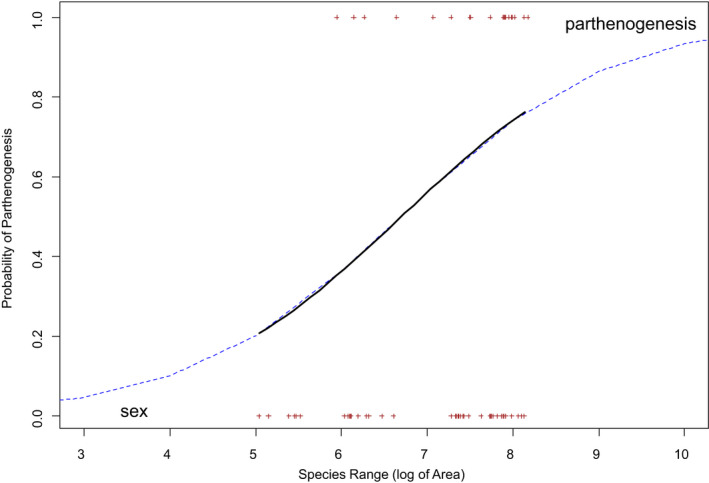
Prediction of the phylogenetic generalized binomial (and linear) model for the relationship between the geographic range size of species and parthenogenetic reproduction (binary data; yes (1) or no (0)). Range size of species showed no phylogenetic signal while reproductive mode did (see text for details). Each point (cross) corresponds to a species. The solid, black line shows the range sizes of the actual data and the dashed line the extrapolation of the model beyond the observed range size of species

The phylogenetic generalized binomial (and linear) model for the binary data with parthenogenesis yes (1) or no (0), including our reconstructed phylogenetic tree as input for the covariance matrix of the residuals, showed a positive relationship between species range size and the probability that a species is parthenogenetic (*Z*‐score: 2.059, *p* = .039). The model predicted that the range size is positively correlated with the probability of being parthenogenetic (Figure 3).

## 4 DISCUSSION

The findings of our study indicate that parthenogenetic oribatid mite species occupy broader geographic ranges than sexual species, although few sexual species have larger range sizes than parthenogenetic species. The results therefore argue against our null hypothesis and are consistent with our expectation that parthenogenetic oribatid mite species have a broader ecological range and possess a more generalistic genotype than sexual species. This conclusion, of course, is based on the assumption that geographic range serves as a proxy for the degree of generalism/specialization (see below). Overall, the results support the general‐purpose genotype (GPG) hypothesis and indicate that, over long evolutionary periods of time, broadly adapted generalistic genotypes with wide niches will persist longer than specialized genotypes with narrow niches (Lynch, 1984). This corroborates previous studies on the genetic variance at the population level in sexual and parthenogenetic oribatid mites (Heethoff et al., 2007; Lienhard & Krisper, 2021; Schäffer et al., 2019; Von Saltzwedel et al., 2014).

Parthenogenetic oribatid mites therefore may be viewed as “Jack‐of‐all‐traits,” i.e., being characterized by broad niches and ranges, and nevertheless being evolutionary successful (Remold, 2012). Interestingly, a similar pattern also exists in asexual parasites that often have wide host ranges and wide distribution range sizes (Gibson, 2019) indicating that the pattern observed in oribatid mites is not limited to soil organisms (Hanley et al., 1994; Van Doninck et al., 2002).

Our assumption that the range size of species is a proxy for the degree of generalism with species with larger ranges being more generalistic, however, may not uniformly apply since widespread species may also occupy specific niches that occur worldwide (Kearney et al., 2003; Parker, 1979, 2002), e.g., oribatid mite species of the taxa Ameronothridae and Fortuyniidae occur all over the world but almost exclusively in coastal ecosystems (Pfingstl, 2013, 2017). As our study covered a wide range of species of different ecosystems and was controlled for the phylogenetic signal of the mode of reproduction, our conclusion that species with larger range sizes are more likely to reproduce via parthenogenesis than via sexual reproduction is likely to hold. In case of oribatid mites and other soil animals, trophic generalism is the rule due to the limited ability to locate and reach new resources and limited dispersal (Cordes et al., 2022; Digel et al., 2014; Erktan et al., 2020; Krause et al., 2021; Lehmitz et al., 2011; Scheu, 2002; Scheu & Setälä, 2002). Typical species that occur in broad geographical ranges and also occupy many different habitats and niches are, e.g., *Playtnothrus peltifer*, *Oppiella nova*, and species of the genera *Tectocepheus* and *Nanhermannia* (Magilton et al., 2019; Meyer et al., 2022; Schneider et al., 2004; Von Saltzwedel et al., 2014).

One difficulty when using the generalist–specialist dichotomy in ecological and evolutionary studies is that there is no universally accepted definition of generalists and specialists in ecology because species can be generalist and specialist at the same time with respect to different environmental conditions and traits (Richmond et al., 2005). In general, however, species occupying small range sizes are likely to be adapted to local habitat conditions and resources due to ecological speciation (Bush & Butlin, 2004; Nosil, 2002). Intriguingly, a species that is, for example, globally distributed might consist of a range of genotypes, which are locally specialized, whereas at species level, it appears to be a generalist. More detailed studies on the genetic structure of sexual and parthenogenetic oribatid mite species therefore are needed to judge whether this limits our conclusions.

The correlation between range sizes and parthenogenetic reproduction, and the consistency with the GPG model fits well with the old age of oribatid mites. According to the fossil record and to molecular clock estimations, oribatid mites emerged in the Cambrian or earlier (Schaefer et al., 2010; Schaefer & Caruso, 2019), with some species having changed little for tens of millions of years (Heethoff et al., 2007; Schäffer et al., 2010). Presumably, for surviving such long geological times old parthenogenetic species of oribatid mites had to comprise broadly adapted genotypes. However, recent parthenogenetic mite species with narrow niches and small range sizes also exist, and the range sizes of parthenogenetic species in fact vary strongly. Further, even rather young parthenogenetic oribatid mite species may be very widespread and this may apply, e.g., to *Oppiella nova* and the false spider mite *Brevipalpus phoenicis* (Groot et al., 2005; Von Saltzwedel et al., 2014).

As our results suggest that parthenogenetic oribatid mite species have more generalistic genotypes and are adapted to a wider range of environmental conditions than sexual species they are likely to have a greater potential for being invasive (Andersen et al., 2012; Oplaat & Verhoeven, 2015; but see Drown et al., 2011). For plants, there is increasing evidence that species with generalistic genotypes indeed are vigorous invaders (Richards et al., 2006; Yu & He, 2021). However, this hypothesis needs further testing. Our results are based on a relatively large but still limited dataset. Especially the phylogenetic relationship between the wide range of oribatid mite lineages included and the estimate of the range size of individual species need to be improved. However, as we included representatives of most oribatid families and lineages, we are confident that the results are robust.

Overall, the results suggest that parthenogenetic oribatid mite species on average have larger range sizes than sexual species, with this relationship being independent of phylogenetic relatedness. The more pronounced generalism in parthenogenetic oribatid mites, as indicated by wider ranges, is likely to be related to the adaptation to a wider range of environmental factors but may also reflect the ability to use a wider range of food resources. In fact, parthenogenetic oribatid mite species are more frequent at high latitudes (Maraun et al., 2019) where environmental conditions fluctuate more than at lower latitudes favoring generalistic genotypes. Furthermore, parthenogenetic species also often live as primary decomposers, feeding on resources that are universally available in ample supply, whereas sexual taxa may feed on more specific resources varying more in space and time. This is supported by stable isotope analysis indicating that oribatid mite species with narrow trophic niches, such as lichen feeders (e.g., *Mycobates* spp., *Jugatala* spp., *Cymberemaeus cymba*, *Carabodes labyrinthicus*) and moss feeders (e.g., *Melanozetes mollicomus*) and species feeding on marine algae (e.g., *Ameronothrus schneideri*, *Hermannia pulchella*, *Zachvatkinibates quadrivertex*) are predominantly sexual (Bluhm et al., 2015; Haynert et al., 2017; Maraun et al., 2011; Winter et al., 2018). Clearly, our data cannot resolve all the factors that can contribute to the relationship that links species range to reproductive mode but can demonstrate that statistical relationship and offer possible hypotheses to explain it. Testing those new hypotheses will require more experimental data and evidence in the future.

In summary, we provided evidence that parthenogenetic oribatid mite species have a widely adapted GPG and may be viewed as “Jack‐of‐all‐trades.” As parthenogenetic and sexual oribatid mite species co‐exist in many habitats and specialist species typically outcompete generalist species the question arises why sexual species do not displace parthenogenetic species. However, as stressed by Scheu and Drossel (2007) and Song et al. (2011) such displacement is unlikely if resources are in ample supply or are regrowing or being replenished fast. In fact, this is consistent with the dominance of parthenogenetic species in forests with thick litter layers and at high latitude ecosystems, whereas sexuals dominate in systems with less and more variable resource availability such as tropical habitats and the bark of trees (Maraun et al., 2019). Essentially, it needs to be investigated if widely distributed parthenogenetic species comprise few or many genotypes or cryptic species using molecular tools, allowing to elucidate if the success of parthenogenetic oribatid mite species is based on many narrowly adapted genotypes or few widely adapted ones. Ultimately, these studies may allow us to resolve if sex increases or decreases genetic variation in the long term (Gorelick & Heng, 2010).

## AUTHOR CONTRIBUTIONS

**Mark Maraun:** Conceptualization (equal); data curation (equal); formal analysis (equal); investigation (equal); methodology (equal); project administration (equal); resources (equal); software (equal); supervision (equal); validation (equal); visualization (equal); writing – original draft (equal); writing – review and editing (equal). **Paul S. P. Bischof:** Conceptualization (equal); investigation (equal); methodology (equal); validation (equal); visualization (equal); writing – original draft (equal). **Finn L. Klemp:** Formal analysis (equal); investigation (equal); methodology (equal); validation (equal). **Jule Pollack:** Formal analysis (equal); methodology (equal); validation (equal); visualization (equal). **Linnea Raab:** Formal analysis (equal); investigation (equal); methodology (equal); validation (equal). **Jan Schmerbach:** Formal analysis (equal); investigation (equal); methodology (equal); validation (equal). **Ina Schaefer:** Formal analysis (equal); investigation (equal); methodology (equal); resources (equal); software (equal); supervision (equal); validation (equal). **Stefan Scheu:** Conceptualization (equal); formal analysis (equal); funding acquisition (equal); methodology (equal); project administration (equal); supervision (equal); validation (equal); visualization (equal); writing – review and editing (equal). **Tancredi Caruso:** Conceptualization (equal); formal analysis (equal); investigation (equal); methodology (equal); software (equal); validation (equal); writing – review and editing (equal).

## CONFLICT OF INTEREST

The authors declare that they have no conflict of interest.

## Supporting information


Appendix S1

Appendix S2

Appendix S3

Appendix S4

## Data Availability

Data are available from the Dryad Digital Repository (https://doi.org/10.5061/dryad.z612jm6d8).
