# Supplementary material for: “Jack‐of‐all‐trades” is parthenogenetic
Source: Ecol Evol. 2022 Jun 23;12(6):e9036. doi: 10.1002/ece3.9036 (PMC9219104; doi:10.1002/ece3.9036)
Supplement: Supplementary file 1 — Appendix S1 [file ECE3-12-e9036-s001.pdf]

Appendix; Table1. The geographic regions of the occurence of oribatid mites according to Subías (2022), their size (km²) and the internet source from which the respective size of each region was extracted.

Region (Subías 2022)	Region (English)	Area (km²)
África centrooriental	Africa (Central Eastern)	4,350,015
África occidental	Africa (Western)	6,409,409
África oriental	Africa (Eastern)	7,670,709
Alaska	U.S.A. (Alaska)	1,717,854
Alemania	Germany	348,672
Alpine	Alpine bioms	3,560,000
Angola	Angola	1,246,700
Antártica	Antarctica	14,000,000
Argentina	Argentina	2,736,690
Arunachal Pradesh	India (Arunachal Pradesh)	83,743
Asia centrooccidental	Asia (Western-Central)	1,334,484
Australia	Australia (country)	7,682,300
Australiana	Australasia (realm)	7,700,000
Azores	Azores	2,351
Bali	Bali	5,780
Bengala occidental	India (West Bengal)	88,752
Bolivia	Bolivia	1,083,301
Boreal	Boreal Zone	19,500,000
Boreal (excepto Paleártica oriental)	Boreal Zone (except Eastern Paleartic)	6,580,586
Boreoalpina	Boreal & Alpine zone	78,249,340
Boreoalpina: Euroatlántica	Boreoalpine zone	385,207
Borneo	Borneo	751,936
Brasil	Brasil	8,358,140
Bután	Butan	38,394
Cachemira	Kashmir (region)	222,000
California	U.S.A. (California)	163,696
Camboya	Cambodia	181,035
Camerún	Cameroon	472,710
Canadá	Canada	9,093,507

Canarias	Canary Islands	7,492
Carolina del Norte	U.S.A. (North Carolina)	139,389
Cáucaso	Caucasus	400,000
Ceilán/Sri Lanka	Sri Lanka	64,630
Centroamérica	Central America (excl. Mexico)	521,876
Chad	Chad	1,259,200
Chile	Chile	743,812
China suroriental	China (South-Eastern)	1,837,108
Colombia	Colombia	1,038,700
Congo	Congo (DRC)	2,344,858
Corea	Korean Peninsula	213,019
Cosmopolita	Cosmopolitan	148,940,000
Costa Rica	Costa Rica	51,100
Crimea	Crimea	26,844
Crozet	Crozet Islands	352
Cuba	Cuba	109,820
Ecuador	Ecuador	283,561
Egipto	Egypt	1,001,449
El Salvador	El Salvador	21,041
Este de Paleártica oriental	Palearctic (Eastern part of the East)	6,011,111
Este de Rusia asiática/ Este de Rusia oriental	Russia (East of Asian)	6,952,600
Este de U.S.A.	U.S.A. (Eastern)	1,791,708
Etiopía	Ethiopia (country)	1,000,000
Etiópica	Afrotropic = Ethiopic (realm)	22,100,000
Europa	Europe	10,180,000
Europa central	Europe (Central)	990,489
Europa centromeridional	Europe (Central-Southern)	294,507
Europa centrooccidental	Europe (Central-Western)	247,622
Europa meridional	Europe (Southern)	1,317,044
Europa occidental/Oeste de Europa	Europe (Western)	1,088,001
Europa: excepto Norte	Europe (excl. Northern)	9,018,924
Europa: Norte	Europe (Northern)	1,161,076
Fiji	Fiji	18,274

Filipinas	Philippines	298,170
Florida	U.S.A. (Florida)	170,304
Galápagos	Galapagos Islands	8,010
Georgia del Sur	South Georgia Islands	3,756
Ghana	Ghana	227,533
Groenlandia	Greenland	2,166,086
Hawái	U.S.A. (Hawaii)	16,625
Himalaya	Himalaya	600,000
Holanda	Holland	7,511
Holártica	Holarctic = Paleartic & Nearctic	77,000,000
Holártica meridional	Holarctic (Southern)	23,927,119
I. Ámsterdam	Amsterdam Island/New Amsterdam	55
I. Mauricio	Mauritius	2,040
I. San Pablo	Saint Paul Island	6
Illinois	U.S.A. (Illinois)	149,998
India	India	3,287,469
Indonesia	Indonesia	1,904,569
Irán	Iran	1,531,595
Islás Británicas	British Isles	307,890
Islas de Pacifico	Pacific Islands	1,252,700
Italia	Italy	301,338
Japón	Japan	377,975
Java	Java	126,700
Kenia	Kenya	569,140
Kerala	India (Kerala)	38,863
Kuriles	Kuril Islands	10,503
La Española: R. Dominicana	Dominican Republic	48,442
Laos	Laos	236,800
Lombok	Lombok	4,725
Luisiana	U.S.A. (Louisiana)	134,264
Malgache	Madagascar	581,540
Marianas	Mariana Islands	477
Mediterránea	Mediterranean Basin	2,085,292

Mediterránea occidental	Mediterranean Basin (Western)	1,390,194
Mediterránea oriental	Mediterranean Basin (Eastern)	695,097
Megalaya	India (Meghalaya)	22,429
Méjico	Mexico	1,972,550
Melanesia	Melanesia	940,000
Michigan	U.S.A. (Michigan)	250,494
Minesota	U.S.A. (Minnesota)	225,171
Mongolia	Mongolia	1,566,000
Neártica	Nearctic	22,900,000
Neártica austral	Nearctic (Southern)	5,893,786
Neártica nororiental	Nearctic (North Eastern)	2,166,086
Neártica occidental	Nearctic (Western)	7,633,333
Neártica oriental	Nearctic (Eastern)	7,633,333
Neártica septentrional	Nearctic (Northern)	11,704,690
Neotropical	Neotropic	19,000,000
Neotropical austral	Neotropic (Southern)	5,067,041
Nepal	Nepal	147,516
Nigeria	Nigeria	923,768
Noreste China	China (North-Eastern)	793,300
Noreste de India	India (North-East)	262,230
Noreste de Oriental	Oriental (North-Eastern)	3,099,270
Noroeste de Siberia	Russia (Northwest of Siberia)	788,932
Norte de India	India (North)	1,421,000
Norte de Neotropical	Neotropic (Northern)	905,400
Norte de Oriental	Oriental (Northern)	4,070,156
Nueva Guinea	New Guinea	786,000
Nueva York	U.S.A. (New York)	141,300
Nueva Zelanda	New Zealand	264,537
Nuevas Hébridas [Vanuatu]	Vanuatu	12,190
Nuevo Méjico	U.S.A. (New Mexico)	314,915
Oeste de Siberia	Russia (Western Siberia)	1,714,984
Oriental	Oriental = Indomalaya (realm)	7,500,000
Orissa	India (Odisha)	155,707

Paleártica	Palearctic	54,100,000
Paleártica (excepto Este)	Palearctic (excl. East)	36,066,667
Paleártica centromeridional	Palearctic (Southern Central)	6,011,111
Paleártica meridional	Palearctic (Southern)	18,033,333
Paleártica occidental	Palearctic (Western)	18,033,333
Paleártica occidental (excepto Norte)	Palearctic (Western except North)	12,022,222
Paleártica oriental	Palearctic (Eastern)	18,033,333
Paleártica oriental (excepto Norte)	Palearctic (Eastern excl. North)	12,022,222
Paleártica oriental meridional	Palearctic (South of Eastern)	6,011,111
Paleártica oriental: excepto Este	Palearctic East (excl. East)	12,022,222
Panamá	Panama	75,417
Pantropical	Pantropial	27,604,311
Pantropical excepto Australiana	Pantropical (excl. Australiana)	40,900,000
Pantropical excepto Etiopica	Pantropical (excl. Afrotropic)	18,596,756
Pantropical y Subtropical	Pantropical & Subtropical	81,935,554
Paquistan	Pakistan	796,095
Paraguay	Paraguay	406,752
Peninsula Arabiqua	Arabian Peninsula	3,237,500
Península Malaya	Mayala Peninsula	242,364
Pequeñas Antillas	Lesser Antilles	13,887
Perú	Peru	1,279,996
Polinesia	Polynesia	294,000
Polonia	Poland	312,696
Principe Eduardo	Prince Edward Island (South Africa)	45
Reunión	Reunión	2,504
Ruanda	Ruanda	26,338
Sahara occidental	Western Sahara	266,000
Samoa	Samoa	2,842
Santa Helena	St. Helena	123

Senegal	Senegal	192,530
Seychelles	Seychelles	455
Shetland del Sur	South Shetland Islands	4,700
Sicilia	Siciliy	25,426
Sikkim	India (Sikkim)	7,096
Somalia	Somalia	637,657
Subtropical	Subtropical	54,331,243
Sudáfrica	South Africa (country)	1,214,470
Sudamérica	South America	17,843,000
Suiza	Switzerland	41,285
Sumatra	Sumatra	473,481
Sur de Siberia	Russia (Southern Siberia)	2,572,477
Sureste de U.S.A.	U.S.A. (South-Eastern)	1,504,355
Suroeste de Siberia	Russia (South Western Siberia)	774,846
Tahití	Tahiti	1,044
Tailandia	Thailand	513,115
Taiwan	Taiwan	36,197
Tanzania	Tanzania	945,087
Tejas	U.S.A. (Texas)	695,621
Tonga	Tonga	750
Tripura	India (Tripura)	10,492
Tristan da Cunha	Tristan da Cunha	180
Túnez	Tunisia	163,610
U.S.A.	U.S.A.	9,826,675
U.S.A. centrooriental	U.S.A. (Eastern-Central)	2,870,721
U.S.A. meridional	U.S.A. (Southern)	2,108,407
U.S.A. oriental	U.S.A. (Eastern)	2,276,528
Uruguay	Uruguay	176,215
Uttar Pradesh	India (Uttar Pradesh)	240,928
Venezuela	Venezuela	912,050
Vietnam	Vietnam	331,212

Virginia	U.S.A. (Virginia)	110,785
Zambia	Zambia	752,618

* 'estimated based on previously defined data' means that some areas were calculated from previously

Körner, C., Urbach. D., Paulsen. J. (2021) Mountain definitions and their consequences. Alpine Botany 13

Subías, L. S. (2022). Listado sistemático, sinonímico y biogeográfico de los ácaros oribátidos (Acariforme:

Testolin, R., Attorre, F., Jiménez-Alfaro, B. (2020) Global distribution and bioclimatic characterization of a

, their English translation,

Source
https://es.wikipedia.org/wiki/África_Oriental
https://es.wikipedia.org/wiki/África_Occidental
https://es.wikipedia.org/wiki/Yibuti
https://de.wikipedia.org/wiki/Alaska
https://de.wikipedia.org/wiki/Deutschland
Testolin et al. (2021) Ecography. doi.org/10.1111/ecog.05012
https://de.wikipedia.org/wiki/Angola
https://de.wikipedia.org/wiki/Antarktika
https://en.wikipedia.org/wiki/Argentina
https://de.wikipedia.org/wiki/Arunachal_Pradesh
https://en.wikipedia.org/wiki/Central_Asia
https://en.wikipedia.org/wiki/Australia
https://simple.wikipedia.org/wiki/Ecozone
https://de.wikipedia.org/wiki/Azoren
https://de.wikipedia.org/wiki/Bali
https://en.wikipedia.org/wiki/West_Bengal
https://en.wikipedia.org/wiki/Bolivia
https://geohilfe.de/oekozonen-nach-schultz/die-boreale-zone/
https://de.wikipedia.org/wiki/Boreale_Zone
Körner et al. (2021) Alpine Botany. doi.10.1007/s00035-011-0094-4
https://de.wikipedia.org/wiki/Norwegen
https://de.wikipedia.org/wiki/Borneo
https://en.wikipedia.org/wiki/Brazil
https://de.wikipedia.org/wiki/Bhutan
https://de.wikipedia.org/wiki/Kaschmir
https://en.wikipedia.org/wiki/California
https://en.wikipedia.org/wiki/Cambodia
https://en.wikipedia.org/wiki/Cameroon
https://en.wikipedia.org/wiki/Canada

https://de.wikipedia.org/wiki/Kanarische_Inseln
https://de.wikipedia.org/wiki/North_Carolina
https://de.wikipedia.org/wiki/Kaukasus
https://en.wikipedia.org/wiki/Sri_Lanka
https://de.wikipedia.org/wiki/Zentralamerika
https://en.wikipedia.org/wiki/Chad
https://en.wikipedia.org/wiki/Chile
https://de.wikipedia.org/wiki/Regionen_Chinas
https://en.wikipedia.org/wiki/Colombia
https://de.wikipedia.org/wiki/Demokratische_Republik_Kongo
https://en.wikipedia.org/wiki/Korea
sum of all regions - Wikipedia
https://de.wikipedia.org/wiki/Costa_Rica
https://de.wikipedia.org/wiki/Krim
https://de.wikipedia.org/wiki/Crozetinseln
https://en.wikipedia.org/wiki/Cuba
https://de.wikipedia.org/wiki/Ecuador
https://de.wikipedia.org/wiki/Ägypten
https://de.wikipedia.org/wiki/El_Salvador
estimated based on previously defined data*
https://en.wikipedia.org/wiki/Siberia#Geomorphological_regions
https://en.wikipedia.org/wiki/Eastern_Time_Zone
https://en.wikipedia.org/wiki/Ethiopia
https://simple.wikipedia.org/wiki/Ecozone
https://en.wikipedia.org/wiki/Europe
https://en.wikipedia.org/wiki/Central_Europe
https://en.wikipedia.org/wiki/Central_Europe
estimated based on previous defined data
https://es.wikipedia.org/wiki/Europa_meridional
https://es.wikipedia.org/wiki/Europa_Occidental
estimated based on previously defined data*
https://www.laenderdaten.de/geographie/flaeche_staaten.aspx
https://en.wikipedia.org/wiki/Fiji

https://en.wikipedia.org/wiki/Philippines
https://de.wikipedia.org/wiki/Florida
https://de.wikipedia.org/wiki/Galapagosinseln
https://es.wikipedia.org/wiki/Islas_Georgias_del_Sur
https://en.wikipedia.org/wiki/Ghana
https://en.wikipedia.org/wiki/Greenland
https://de.wikipedia.org/wiki/Hawaii
https://es.wikipedia.org/wiki/Himalaya
https://en.wikipedia.org/wiki/Holland
https://en.wikipedia.org/wiki/Holarctic_realm
estimated based on previously defined data*
https://en.wikipedia.org/wiki/Île_Amsterdam
https://en.wikipedia.org/wiki/Mauritius
https://en.wikipedia.org/wiki/Île_Saint-Paul
https://de.wikipedia.org/wiki/Illinois
https://en.wikipedia.org/wiki/Geography_of_India
https://en.wikipedia.org/wiki/Indonesia
https://en.wikipedia.org/wiki/Iran
https://de.wikipedia.org/wiki/Britische_Inseln
https://en.wikipedia.org/wiki/List_of_islands_in_the_Pacific_Ocean
https://de.wikipedia.org/wiki/Italien
https://en.wikipedia.org/wiki/Japan
https://de.wikipedia.org/wiki/Java_(Insel)
https://en.wikipedia.org/wiki/Kenya
https://en.wikipedia.org/wiki/Kerala
https://en.wikipedia.org/wiki/Kuril_Islands
https://es.wikipedia.org/wiki/República_Dominicana
https://de.wikipedia.org/wiki/Laos
https://de.wikipedia.org/wiki/Lombok
https://de.wikipedia.org/wiki/Louisiana
https://en.wikipedia.org/wiki/Madagascar
https://en.wikipedia.org/wiki/Mariana_Islands
https://en.wikipedia.org/wiki/Mediterranean_Basin

estimated based on previously defined data*
estimated based on previously defined data*
https://de.wikipedia.org/wiki/Meghalaya
https://en.wikipedia.org/wiki/Mexico
https://de.wikipedia.org/wiki/Melanesien
https://de.wikipedia.org/wiki/Michigan
https://de.wikipedia.org/wiki/Minnesota
https://en.wikipedia.org/wiki/Mongolia
https://simple.wikipedia.org/wiki/Ecozone
https://de.wikipedia.org/wiki/Liste_der_Bundesstaaten_der_Vereinigten_Staaten, https://de.wikipedia.org/wiki/Mexiko
estimated based on previously defined data*
estimated based on previously defined data*
estimated based on previously defined data*
estimated based on previously defined data*
https://simple.wikipedia.org/wiki/Ecozone
sum of Falkland Islands, Argentina, Chile, Uruguay, Paraguay, Southern Brazil
https://en.wikipedia.org/wiki/Nepal
https://de.wikipedia.org/wiki/Nigeria
https://de.wikipedia.org/wiki/Regionen_Chinas
https://es.wikipedia.org/wiki/Noreste_de_India
https://en.wikipedia.org/wiki/Indomalayan_realm#/media/File:Ecozone_Indomalaya.svg
https://de.wikipedia.org/wiki/Sibirien_(Föderationskreis)
https://en.wikipedia.org/wiki/North_India
https://en.wikipedia.org/wiki/Neotropical_realm
https://en.wikipedia.org/wiki/Indomalayan_realm
https://de.wikipedia.org/wiki/Neuguinea
https://de.wikipedia.org/wiki/New_York_(Bundesstaat)
https://en.wikipedia.org/wiki/New_Zealand
https://de.wikipedia.org/wiki/Vanuatu
https://de.wikipedia.org/wiki/New_Mexico
https://de.wikipedia.org/wiki/Sibirien_(Föderationskreis)
https://simple.wikipedia.org/wiki/Ecozone
https://en.wikipedia.org/wiki/Odisha

https://simple.wikipedia.org/wiki/Ecozone
estimated based on previously defined data*
estimated based on previously defined data*
estimated based on previously defined data*
estimated based on previously defined data*
estimated based on previously defined data*
estimated based on previously defined data*
estimated based on previously defined data*
estimated based on previously defined data*
estimated based on previously defined data*
https://en.wikipedia.org/wiki/Panama
Estimated based on sources: https://worldmap.harvard.edu/maps/19173, https://www.ncbi.nlm.nih.gov/pmc/articles/PMC6207062/, https://de.wikipedia.org/wiki/Datei:Subtropical.png http://koeppen-geiger.vu-wien.ac.at/pics/Geiger_1954_Map.jpg
estimated based on previously defined data*
Estimated based on sources: https://worldmap.harvard.edu/maps/19173, https://www.ncbi.nlm.nih.gov/pmc/articles/PMC6207062/, https://de.wikipedia.org/wiki/Datei:Subtropical.png http://koeppen-geiger.vu-wien.ac.at/pics/Geiger_1954_Map.jpg
estimated based on previously defined data*
https://de.wikipedia.org/wiki/Pakistan
https://de.wikipedia.org/wiki/Paraguay
https://en.wikipedia.org/wiki/Arabian_Peninsula
https://en.wikipedia.org/wiki/Malay_Peninsula
https://de.wikipedia.org/wiki/Kleine_Antillen
https://en.wikipedia.org/wiki/Peru
https://de.wikipedia.org/wiki/Polynesien
https://en.wikipedia.org/wiki/Poland
https://de.wikipedia.org/wiki/Prinz-Edward-Inseln
https://de.wikipedia.org/wiki/Reunion
https://de.wikipedia.org/wiki/Ruanda
https://es.wikipedia.org/wiki/Sahara_Occidental
https://en.wikipedia.org/wiki/Samoa
https://de.wikipedia.org/wiki/St._Helena_(Insel)

https://en.wikipedia.org/wiki/Senegal
https://en.wikipedia.org/wiki/Seychelles
https://en.wikipedia.org/wiki/South_Shetland_Islands
https://de.wikipedia.org/wiki/Sizilien
https://de.wikipedia.org/wiki/Sikkim
https://en.wikipedia.org/wiki/Somalia
Estimated based on sources: https://worldmap.harvard.edu/maps/19173, https://www.ncbi.nlm.nih.gov/pmc/articles/PMC6207062/, https://de.wikipedia.org/wiki/Datei:Subtropical.png http://koeppen-geiger.vu-wien.ac.at/pics/Geiger_1954_Map.jpg
https://en.wikipedia.org/wiki/South_Africa
https://de.wikipedia.org/wiki/Südamerika
https://en.wikipedia.org/wiki/Switzerland
https://de.wikipedia.org/wiki/Sumatra
https://de.wikipedia.org/wiki/Sibirien_(Föderationskreis)
https://es.wikipedia.org/wiki/Sureste_de_Estados_Unidos
https://de.wikipedia.org/wiki/Sibirien_(Föderationskreis)
https://en.wikipedia.org/wiki/Tahiti
https://de.wikipedia.org/wiki/Thailand
https://en.wikipedia.org/wiki/Taiwan
https://de.wikipedia.org/wiki/Tansania
https://de.wikipedia.org/wiki/Texas
https://en.wikipedia.org/wiki/Tonga
https://de.wikipedia.org/wiki/Tripura
https://de.wikipedia.org/wiki/Tristan_da_Cunha
https://es.wikipedia.org/wiki/Túnez
https://de.wikipedia.org/wiki/Vereinigte_Staaten
https://en.wikipedia.org/wiki/Central_Time_Zone
https://en.wikipedia.org/wiki/Subtropics#/media/File:World_map_indicating_tropics_and_subtropics.png
https://en.wikipedia.org/wiki/Geography_of_Michigan
https://de.wikipedia.org/wiki/Uruguay
https://de.wikipedia.org/wiki/Uttar_Pradesh
https://de.wikipedia.org/wiki/Venezuela
https://en.wikipedia.org/wiki/Vietnam

https://de.wikipedia.org/wiki/Virginia
https://en.wikipedia.org/wiki/Zambia

mentioned regions, e.g. Paleártica oriental as one-third of Paleártica

[illegible]1, 213–217

[illegible], Oribatida) del mundo (excepto fósiles) (17a actualización): 537 pp. http://bba.bioucm.es/cont/docs/RO_1.pdf

[illegible]lpine biomes. Ecography 43, 779–788
