# Supplementary material for: “Jack‐of‐all‐trades” is parthenogenetic
Source: Ecol Evol. 2022 Jun 23;12(6):e9036. doi: 10.1002/ece3.9036 (PMC9219104; doi:10.1002/ece3.9036)
Supplement: Supplementary file 2 — Appendix S2 [file ECE3-12-e9036-s002.pdf]

Appendix; table 2: Species names, taxonomic affiliation (family), reproductive mode, reason for the assigment of the reproductive mode with reference, distributio
Area data are sums of the respective regions as reported in the Appendix, Table 1. Full references are given below the table.

**Species names after Subías (2022)**	**Family**	**Reproductive mode**	**Reason for assignment of the reproductive mode**
Achipteria coleoptrata (Linnaeus, 1758)	Achipteriidae	sexual	sex ratio
Achipteria elegans Schweizer, 1956	Achipteriidae	sexual	inference
Achipteria nitens (Nicolet, 1855)	Achipteriidae	sexual	sex ratio
Achipteria sellnicki Hammen, 1952	Achipteriidae	sexual	inference
Acrogalumna longipluma (Berlese, 1904)	Galumnidae	sexual	inference
Acrotritia ardua Koch, 1841	Euphthiracaridae	thelytokous	sex ratio, rearing
Acrotritia duplicata (Grandjean, 1953)	Euphthiracaridae	thelytokous	rearing
Acrotritia hyeroglyphica (Berlese, 1916)	Euphthiracaridae	sexual	inference
Adoristes ovatus (Koch, 1839)	Liacaridae	sexual	sex ratio
Adrodamaeus femoratus (Koch, 1839)	Gymnodamaeidae	sexual	inference
Adrodamaeus siculus (Berlese, 1910)	Gymnodamaeidae	sexual	inference
Adrodamaeus vietnamicus (Ermilov & Anichkin, 2011)	Gymnodamaeidae	sexual	inference
Afronothrus incisivus Wallwork, 1961	Trhypochthoniidae	thelytokous	sex ratio, rearing
Allogalumna indonesiensis Ermilov, Sandmann, Klarner, Widyastuti & Scheu, 2015	Galumnidae	sexual	inference
Allogalumna monodactyla Ermilov & Anichkin, 2014	Galumnidae	sexual	inference
Allogalumna paranovazealandica Ermilov, Sandmann, Klarner, Widyastuti & Scheu, 2015	Galumnidae	sexual	inference
Allonothrus russeolus Wallwork, 1960	Trhypochthoniidae	thelytokous	sex ratio
Allosuctobelba grandis (Paoli, 1908)	Suctobelbidae	thelytokous	inference
Allosuctobelba obtusa (Jacot, 1938)	Suctobelbidae	thelytokous	sex ratio
Allozetes pusillus (Berlese, 1913)	Ceratozetidae	sexual	inference
Amazoppia tricuspidata Balogh & Mahunka, 1969	Ceratoppiidae	sexual	inference
Amerobelba decedens Berlese, 1908	Amerobelbidae	sexual	inference
Amerus polonicus Kulczynski, 1902	Ameridae	sexual	inference
Amerus troisi (Berlese, 1883)	Ameridae	sexual	inference
Ampullobates ecuadoriensis Ermilov, Sandmann, Marian & Maraun, 2013	Hermanniellidae	sexual	inference
Aphelacarus acarinus (Berlese, 1910)	Palaeacaridae	sexual	inference
Apoplophora cristata Mahunka, 1991	Mesoplophoridae	sexual	inference
Archegozetes longisetosus Aoki, 1965	Trhypochthoniidae	thelytokous	sex ratio, rearing

Archoplophora rostralis (Willmann, 1930)	Mesoplophoridae	thelytokous	sex ratio
Arcoppia dechambrierorum (Mahunka, 1983)	Oppiidae	sexual	inference
Arcoppia hammerae Rodríguez & Subías, 1984	Oppiidae	sexual	inference
Arcoppia tripartita (Hammer, 1961)	Oppiidae	sexual	inference
Arcoppia vittata Hammer, 1979	Oppiidae	sexual	inference
Atropacarus phyllophorus (Berlese, 1904)	Phthiracaridae	sexual	inference
Atropacarus striculus (Koch, 1835)	Phthiracaridae	thelytokous	sex ratio
Austrocarabodes arrogans Pérez-Íñigo, 1967	Carabodidae	sexual	inference
Austrocarabodes ensifer (Sellnick, 1931)	Carabodidae	sexual	inference
Autogneta dalecarlica Forsslund, 1947	Autognetidae	sexual	inference
Autogneta longilamellata (Michael, 1885)	Autognetidae	sexual	inference
Autogneta traegardhi Forsslund, 1947	Autognetidae	sexual	inference
Banksinoma lanceolata (Michael, 1885)	Thyrisomidae	sexual	sex ratio
Basilobelba parmata Okayama, 1980	Basilobelbidae	sexual	inference
Basilobelba retiaria (Warburton, 1912)	Basilobelbidae	sexual	inference
Beckiella capitulum Balogh & Mahunka, 1978	Dampfiellidae	sexual	inference
Belba bartoši Winkler, 1955	Belbidae	sexual	inference
Belba meridionalis Bulanova-Zachvatkina, 1962	Belbidae	sexual	inference
Berlesezetes brazilozetoides Balogh & Mahunka, 1981	Microzetidae	sexual	inference
Berlesezetes ornatissimus (Berlese, 1913)	Microzetidae	sexual	inference
Berniniella bicarinata (Paoli, 1908)	Oppiidae	sexual	inference
Berniniella conjuncta (Strenzke, 1951)	Oppiidae	sexual	sex ratio
Berniniella sigma (Strenzke, 1951)	Oppiidae	sexual	sex ratio
Bipassalozetes striatus (Mihelčič, 1955)	Passalozetidae	sexual	inference
Brachioppia cuscensis Hammer, 1961	Oppiidae	sexual	inference
Brachioppia deliciosa Hammer, 1961	Oppiidae	sexual	inference
Brachychthonius berlesei Willmann, 1928	Brachychthoniidae	thelytokous	rearing
Brachychthonius hirtus Moritz, 1976	Brachychthoniidae	thelytokous	inference
Brachychthonius impressus Moritz, 1976	Brachychthoniidae	thelytokous	sex ratio
Brachychthonius pius Moritz, 1976	Brachychthoniidae	thelytokous	inference
Brassiella neonominata Subías, 2004	Caloppiidae	sexual	inference
Bursoplophora insularis Kahwash, Subías & Ruiz, 1989	Protoplophoridae	sexual	inference

Bursoplophora meridionalis Bernini, 1983	Protoplophoridae	sexual	inference
Caleremaeus monilipes (Michael, 1882)	Caleremaeidae	sexual	inference
Camisia biurus (Koch, 1839)	Crotoniidae	thelytokous	inference
Camisia hamulifera Hammer, 1961	Crotoniidae	thelytokous	inference
Camisia horrida (Hermann, 1804)	Crotoniidae	thelytokous	sex ratio
Camisia invenusta (Michael, 1888)	Crotoniidae	thelytokous	sex ratio
Camisia khencensis Hammer, 1961	Crotoniidae	thelytokous	inference
Camisia lapponica (Trägårdh, 1910)	Crotoniidae	thelytokous	inference
Camisia segnis (Hermann, 1804)	Crotoniidae	thelytokous	sex ratio, rearing
Camisia solhoeyi Colloff, 1993	Crotoniidae	thelytokous	inference
Camisia spinifer (Koch, 1836)	Crotoniidae	thelytokous	sex ratio, rearing
Campachipteria fanzagoi (Jacot, 1929)	Achipteriidae	sexual	inference
Campachipteria perproxima (Sellnick, 1931)	Achipteriidae	sexual	inference
Campachipteria petiti (Travé, 1960)	Achipteriidae	sexual	inference
Campachipteria weigmanni (Pérez-Íñigo, 1987)	Achipteriidae	sexual	inference
Carabodes areolatus Berlese, 1916	Carabodidae	sexual	inference
Carabodes coriaceus Koch, 1835	Carabodidae	sexual	inference
Carabodes femoralis (Nicolet, 1855)	Carabodidae	sexual	inference
Carabodes labyrinthicus (Michael, 1879)	Carabodidae	sexual	sex ratio
Carabodes marginatus (Michael, 1884)	Carabodidae	sexual	inference
Carabodes ornatus Štorkán, 1925	Carabodidae	sexual	sex ratio
Carabodes reticulatus Berlese, 1913	Carabodidae	sexual	inference
Carabodes rugosior Berlese, 1916	Carabodidae	sexual	inference
Carabodes samoensis J. & P. Balogh, 1986	Carabodidae	sexual	inference
Carabodes subarcticus Trägårdh, 1902	Carabodidae	sexual	inference
Carabodes willmanni Bernini, 1975	Carabodidae	sexual	inference
Caucasiozetes frankeae Ermilov & Anichkin, 2011	Microzetidae	sexual	inference
Cavaecarabodes orientalis (Mahunka, 1987)	Carabodidae	sexual	inference
Cavernocepheus monstruosus Balogh & Mahunka, 1969	Dampfiellidae	sexual	inference
Cepheus cepheiformis (Nicolet, 1855)	Cepheidae	sexual	inference
Cepheus dentatus (Michael, 1888)	Cepheidae	sexual	inference
Cepheus latus Koch, 1835	Cepheidae	sexual	inference
Ceratoppia bipilis (Hermann, 1804)	Ceratoppiidae	sexual	inference

Ceratoppia quadridentata (Haller, 1882)	Ceratoppiidae	sexual	inference
Ceratoppia sexpilosa Willmann, 1938	Ceratoppiidae	sexual	inference
Ceratoppia sphaerica (C.L. Koch, 1879)	Ceratoppiidae	sexual	inference
Ceratorchestes globosus Balogh & Mahunka, 1969	Ceratoppiidae	sexual	inference
Ceratozetes cuspidatus Jacot, 1939	Ceratozetidae	thelytokous	sex ratio
Ceratozetes gracilis (Michael, 1884)	Ceratozetidae	sexual	sex ratio
Ceratozetes mediocris Berlese, 1908	Ceratozetidae	sexual	inference
Ceratozetes pacificus Behan-Pelletier, 1984	Ceratozetidae	sexual	inference
Ceratozetes peritus Grandjean, 1951	Ceratozetidae	sexual	sex ratio
Chamobates birulai (Kulczynski, 1902)	Chamobatidae	sexual	inference
Chamobates cuspidatus (Michael, 1884)	Chamobatidae	sexual	sex ratio
Chamobates pusillus (Berlese, 1895)	Chamobatidae	sexual	inference
Chamobates subglobulus (Oudemans, 1900)	Chamobatidae	sexual	sex ratio
Chamobates voigtsii (Oudemans, 1902)	Chamobatidae	sexual	inference
Chistyakovella insolita Ermilov, Aoki & Anichkin, 2013	Carabodidae	sexual	inference
Coartobelba loksai (Balogh & Mahunka, 1981)	Suctobelbidae	thelytokous	inference
Cosmochthonius lanatus (Michael, 1885)	Cosmochthoniidae	sexual	inference
Cosmochthonius plumatus Berlese, 1910	Cosmochthoniidae	sexual	inference
Cosmohermannia robusta (Aoki, 1994)	Nanhermanniidae	thelytokous	inference
Cosmozetes vermiculatus (Balogh & Mahunka, 1980)	Microzetidae	sexual	inference
Cryptacarus promecus Grandjean, 1950	Lohmanniidae	thelytokous	sex ratio
Cryptoplophora abscondita Grandjean, 1932	Protoplophoridae	sexual	inference
Cryptoppia mahunkai (Wang & Li, 1997)	Oppiidae	sexual	inference
Ctenacarus araneola (Grandjean, 1932)	Ctenacaridae	sexual	inference
Cultroribula berolina Weigmann, 2006	Astegistidae	thelytokous	inference
Cultroribula bicultrata (Berlese, 1905)	Astegistidae	thelytokous	sex ratio
Cultroribula divergens Jacot, 1939	Astegistidae	thelytokous	sex ratio
Cultroribula juncta (Michael, 1885)	Astegistidae	thelytokous	inference
Cultroribula lata Aoki, 1961	Astegistidae	thelytokous	inference
Cultroribula zicsii Balogh & Mahunka, 1981	Astegistidae	thelytokous	inference
Cycloppia restata (Aoki, 1963)	Oppiidae	sexual	inference
Cymbaeremaeus cymba (Nicolet, 1855)	Cymbaeremaeidae	sexual	inference
Cyrthermannia florens Balogh & Mahunka, 1980	Nanhermanniidae	thelytokous	inference

Cyrthermannia tuberculata Balogh, 1958	Nanhermanniidae	thelytokous	inference
Cyrthermannia vicinicornuta Aoki, 1965	Nanhermanniidae	thelytokous	inference
Damaeobelba minutissima (Sellnick, 1929)	Damaeidae	thelytokous	sex ratio
Damaeolus ornatissimus Csiszár, 1962	Damaeolidae	sexual	inference
Damaeus angustipes (Banks, 1905)	Damaeidae	sexual	inference
Damaeus bituberculatus (Kulczynski, 1902)	Damaeidae	sexual	inference
Damaeus boreus Bulanova-Zachvatkina, 1957	Damaeidae	sexual	inference
Damaeus clavipes (Hermann, 1804)	Damaeidae	sexual	inference
Damaeus fageti Bulanova-Zachvatkina, 1957	Damaeidae	sexual	inference
Damaeus flagellatus Wang, 1994	Damaeidae	sexual	inference
Damaeus flagelloides (Norton, 1979)	Damaeidae	sexual	inference
Damaeus onustus Koch, 1841	Damaeidae	sexual	sex ratio
Damaeus riparius Nicolet, 1855	Damaeidae	sexual	sex ratio
Damaeus tecticola Michael, 1888	Damaeidae	sexual	inference
Dendrohermannia monstruosa (Aoki, 1977)	Nanhermanniidae	thelytokous	inference
Diapterobates humeralis (Hermann, 1804)	Humerobatidae	sexual	inference
Diapterobates notatus (Thorell, 1871)	Humerobatidae	sexual	inference
Discoppia cylindrica (Pérez-Íñigo, 1965)	Oppiidae	sexual	inference
Dissorhina ornata (Oudemans, 1900)	Oppiidae	sexual	sex ratio
Dolicheremaeus aokii (Balogh & Mahunka, 1967)	Tetracondylidae	sexual	inference
Dolicheremaeus bugiamapensis Ermilov, Anichkin & Wu, 2012	Tetracondylidae	sexual	inference
Dolicheremaeus contactus Ermilov & Anichkin, 2013	Tetracondylidae	sexual	inference
Dolicheremaeus donacunarensis Ermilov & Anichkin, 2014	Tetracondylidae	sexual	inference
Dolicheremaeus dorni (Balogh, 1937)	Tetracondylidae	sexual	inference
Dolicheremaeus dwalteri Ermilov & Anichkin, 2014	Tetracondylidae	sexual	inference
Dolicheremaeus sumatranus Mahunka, 1989	Tetracondylidae	sexual	inference
Dolicheremaeus variolatus Mahunka, 1989	Tetracondylidae	sexual	inference
Dolicheremaeus variolobatus Hammer, 1981	Tetracondylidae	sexual	inference
Edwardzetes armatus (Hammer, 1958)	Ceratozetidae	sexual	inference
Edwardzetes edwardsi (Nicolet, 1855)	Ceratozetidae	sexual	inference
Eobrachychthonius latior (Berlese, 1910)	Brachychthoniidae	thelytokous	inference
Eohypochthonius salicifolius Hammer, 1979	Hypochthoniidae	thelytokous	inference
Epieremulus granulatus (Balogh & Mahunka, 1979)	Caleremaeidae	sexual	inference

Epieremulus longiseta (P. Balogh, 1988)	Caleremaeidae	sexual	inference
Epilohmannia crassisetosa Ermilov & Anichkin, 2012	Epilohmanniidae	sexual	inference
Epilohmannia cylindrica (Berlese, 1904)	Epilohmanniidae	thelytokous	sex ratio
Epilohmannia minuta Berlese, 1920	Epilohmanniidae	thelytokous	inference
Epilohmannoides wallworki Hammer, 1981	Epilohmanniidae	sexual	inference
Eremaeus granulatus Mihelčič, 1955	Eremaeidae	sexual	inference
Eremaeus hepaticus Koch, 1835	Eremaeidae	sexual	inference
Eremella induta Berlese, 1913	Eremellidae	sexual	inference
Eremobelba bellicosa Balogh & Mahunka, 1967	Ctenobelbidae	sexual	inference
Eremobelba breviseta Balogh, 1968	Ctenobelbidae	sexual	inference
Eremobelba comteae Mahunka, 1988	Ctenobelbidae	sexual	inference
Eremobelba gracilior Berlese, 1908	Ctenobelbidae	sexual	sex ratio
Eremobelba pectinigera Berlese, 1908	Ctenobelbidae	sexual	inference
Eremulus baliensis Hammer, 1982	Caleremaeidae	sexual	inference
Eremulus densus Hammer, 1979	Caleremaeidae	sexual	inference
Eremulus nigrisetosus Hammer, 1958	Caleremaeidae	sexual	inference
Eueremaeus silvestris (Forsslund, 1956)	Eremaeidae	sexual	inference
Eulohmannia ribagai (Berlese, 1910)	Eulohmanniidae	thelytokous	sex ratio
Eupelops acromios (Hermann, 1804)	Phenopelopidae	sexual	inference
Eupelops claviger (Berlese, 1916)	Phenopelopidae	sexual	inference
Eupelops major (Hull, 1914)	Phenopelopidae	sexual	inference
Eupelops occultus (Koch, 1835)	Phenopelopidae	sexual	sex ratio
Eupelops plicatus (Koch, 1835)	Phenopelopidae	sexual	sex ratio
Eupelops subuliger (Berlese, 1916)	Phenopelopidae	sexual	inference
Eupelops torulosus (Koch, 1839)	Phenopelopidae	sexual	inference
Euphthiracarus cribrarius (Berlese, 1904)	Euphthiracaridae	sexual	inference
Eupterotegaeus dentatus Sitnikova, 1979	Cepheidae	sexual	inference
Eurostocepheus aquilinus Aoki, 1965	Otocepheidae	sexual	inference
Euzetes globulus (Nicolet, 1855)	Ceratozetidae	sexual	inference
Fuscozetes fuscipes (Koch, 1844)	Ceratozetidae	sexual	inference
Fuscozetes setosus (Koch, 1839)	Ceratozetidae	sexual	sex ratio
Galumna alata (Hermann, 1804)	Galumnidae	sexual	inference

Galumna areticulata Ermilov, Sandmann, Klarner, Widyastuti & Scheu, 2015	Galumnidae	sexual	inference
Galumna bidentatirostris Ermilov, Sandmann, Klarner, Widyastuti & Scheu, 2015	Galumnidae	sexual	inference
Galumna calva Starý, 1997	Galumnidae	sexual	inference
Galumna corpuzrarosae Ermilov, Sandmann, Klarner, Widyastuti & Scheu, 2015	Galumnidae	sexual	inference
Galumna dimorpha Krivolutskaja, 1952	Galumnidae	sexual	inference
Galumna dispar Willmann, 1932	Galumnidae	sexual	inference
Galumna dongnaiensis Ermilov & Anichkin, 2013	Galumnidae	sexual	inference
Galumna flabellifera Hammer, 1958	Galumnidae	sexual	inference
Galumna indonesica Ermilov, Sandmann, Klarner, Widyastuti & Scheu, 2015	Galumnidae	sexual	inference
Galumna ithacensis (Jacot, 1929)	Galumnidae	sexual	sex ratio
Galumna khoii Mahunka, 1989	Galumnidae	sexual	inference
Galumna lanceata (Oudemans, 1900)	Galumnidae	sexual	sex ratio
Galumna levisensilla Emilov & Anichkin, 2010	Galumnidae	sexual	inference
Galumna mikoi Ermilov, Sandmann, Klarner, Widyastuti & Scheu, 2015	Galumnidae	sexual	inference
Galumna miniporosa Ermilov, Starý, Sandmann & Maraun, 2013	Galumnidae	sexual	inference
Galumna paracalcicola Ermilov & Anichkin, 2014	Galumnidae	sexual	inference
Galumna parakazakhstan i Ermilov & Anichkin, 2014	Galumnidae	sexual	inference
Galumna pseudokhoii Ermilov & Anichkin, 2011	Galumnidae	sexual	inference
Galumna sabahna Mahunka, 1995	Galumnidae	sexual	inference
Galumna sumatrensis Ermilov, Sandmann, Klarner, Widyastuti & Scheu, 2015	Galumnidae	sexual	inference
Galumnella geographica Mahunka, 1995	Galumnellidae	sexual	inference
Galumnella microporosa Ermilov & Anichkin, 2011	Galumnellidae	sexual	inference
Galumnopsis reducta (Mahunka, 1995)	Galumnellidae	sexual	inference
Gehypochthonius rhadamanthus Jacot, 1936	Gehypochthoniidae	thelytokous	sex ratio
Gigantoppia zryanini Ermilov & Anichkin, 2011	Granuloppiidae	sexual	inference
Gittella flagellata (Mahunka, 1983)	Oppiidae	sexual	inference
Gittella variabilis Ermilov, Sandmann, Marian & Maraun, 2013	Oppiidae	sexual	inference
Globogalumna biporosa Ermilov & Anichkin, 2012	Galumnidae	sexual	inference
Gozmanyina majestus (Marshall & Reeves, 1970)	Trichthoniidae	thelytokous	sex ratio
Graptoppia sundensis (Hammer, 1979)	Oppiidae	sexual	inference
Gustavia fusifer (Koch, 1841)	Gustaviidae	sexual	inference
Gustavia microcephala (Nicolet, 1855)	Gustaviidae	sexual	inference
Gymnodamaeus bicostatus (Koch, 1835)	Gymnodamaeidae	sexual	sex ratio

Hammerella parasufflata Ermilov, Sandmann, Marian & Maraun, 2013	Granuloppiidae	sexual	inference
Haplochthonius sanctaeluciae Bernini, 1973	Haplochthoniidae	thelytokous	inference
Hellenamerus ionicus Mahunka, 1974	Amerobelbidae	sexual	inference
Hemileius initialis (Berlese, 1908)	Hemileiidae	sexual	inference
Hemileius perforatoides (Hammer, 1979)	Hemileiidae	sexual	inference
Heminothrus ornatissimus (Berlese, 1910)	Crotoniidae	thelytokous	sex ratio, rearing
Heminothrus targionii (Berlese, 1885)	Crotoniidae	thelytokous	sex ratio
Hermannia gladiata (Aoki, 1965)	Hermanniidae	sexual	inference
Hermannia convexa (Koch, 1839)	Hermanniidae	sexual	inference
Hermannia gibba (Koch, 1839)	Hermanniidae	sexual	sex ratio
Hermannia reticulata Thörell, 1871	Hermanniidae	sexual	inference
Hermannia similis (Balogh & Mahunka, 1967)	Hermanniidae	sexual	inference
Hermannia subglabra Berlese, 1910	Hermanniidae	sexual	inference
Hermanniella dolosa Grandjean, 1931	Hermanniellidae	sexual	inference
Hermannobates monstruosus Hammer, 1961	Hermanniellidae	sexual	inference
Heterobelba galerulata Berlese, 1913	Heterobelbidae	sexual	inference
Heterobelba oxapampensis Beck, 1962	Heterobelbidae	sexual	inference
Heteroleius longissimus Balogh & Mahunka, 1966	Hemileiidae	sexual	inference
Hoplophorella cucullata (Ewing, 1909)	Phthiracaridae	sexual	inference
Hoplophorella vitrina (Berlese, 1913)	Phthiracaridae	sexual	inference
Hoplophthiracarus illinoisensis (Ewing, 1909)	Phthiracaridae	sexual	inference
Hypocepheus mirabilis Krivolutsky, 1971	Cepheidae	sexual	inference
Hypochthoniella minutissima (Berlese, 1903)	Eniochthoniidae	thelytokous	sex ratio
Hypochthonius luteus Oudemans, 1917	Hypochthoniidae	thelytokous	sex ratio
Hypochthonius rufulus C.L. Koch, 1835	Hypochthoniidae	thelytokous	sex ratio
Idiozetes javensis Hammer, 1979	Eremaeozetidae	sexual	inference
Indoribates microsetosus Ermilov & Anichkin, 2011	Haplozetidae	sexual	inference
Indoribates paraminimicoma (Ermilov, Bayartogtokh, Sandmann, Marian & Maraun, 2013)	Haplozetidae	sexual	inference
Indoribates punctulatus (Sellnick, 1925)	Haplozetidae	sexual	inference
Indoribates vindobonensis (Willmann, 1935)	Haplozetidae	sexual	inference
Insculptoppia insculpta (Paoli, 1908)	Oppiidae	sexual	inference
Jacotella reticulata Ruiz, Kahwash & Subías, 1990	Gymnodamaeidae	sexual	sex ratio
Javacarus jocelynae Judson, 1991	Lohmanniidae	thelytokous	inference

Javacarus kuehnelti Balogh, 1961	Lohmanniidae	thelytokous	inference
Joelia fiorii (Coggi, 1898)	Oribatellidae	sexual	inference
Karenella acuta (Csiszár, 1961)	Oppiidae	sexual	inference
Kokoppia dudichi (Balogh, 1982)	Oppiidae	sexual	inference
Kuklosuctobelba claviseta (Hammer, 1961)	Suctobelbidae	thelytokous	inference
Lamellobates misella (Berlese, 1910)	Oribatellidae	sexual	inference
Lamellobates molecula (Berlese, 1916)	Oribatellidae	sexual	inference
Lauritzenia minimicoma (Beck, 1964)	Haplozetidae	sexual	inference
Lauroppia falcata (Strenzke, 1951)	Oppiidae	sexual	sex ratio
Lauroppia fallax (Paoli, 1908)	Oppiidae	sexual	inference
Lauroppia maritima (Willmann, 1928)	Oppiidae	sexual	inference
Lauroppia similifallax Subías & Mínguez, 1986	Oppiidae	sexual	inference
Leptogalumna ciliata Balogh, 1960	Galumnidae	sexual	inference
Liacarus acutus Pschorn-Walcher, 1951	Liacaridae	sexual	inference
Liacarus brevilamellatus Mihelčič, 1955	Liacaridae	sexual	inference
Liacarus koeszegiensis Balogh, 1943	Liacaridae	sexual	inference
Liacarus nitens (Gervais, 1844)	Liacaridae	sexual	inference
Liacarus subterraneus (Koch, 1844)	Liacaridae	sexual	sex ratio
Liacarus xylariae (Schrank, 1803)	Liacaridae	sexual	inference
Licneremaeus embeyisztini Mahunka, 1980	Licneremaeidae	sexual	inference
Licneremaeus licnophorus (Michael, 1882)	Licneremaeidae	sexual	inference
Licnodamaeus costula Grandjean, 1931	Licnodamaeidae	sexual	inference
Licnozetes granulatus (Balogh & Mahunka, 1969)	Microzetidae	sexual	inference
Liebstadia humerata Sellnick, 1928	Liebstadiidae	sexual	inference
Liebstadia longior (Berlese, 1908)	Liebstadiidae	sexual	inference
Liebstadia similis (Michael, 1888)	Liebstadiidae	sexual	sex ratio
Liochthonius alpestris (Forsslund, 1958)	Brachychthoniidae	thelytokous	inference
Liochthonius brevis (Michael, 1888)	Brachychthoniidae	thelytokous	inference
Liochthonius dilutus Moritz, 1976	Brachychthoniidae	thelytokous	inference
Liochthonius evansi (Forsslund, 1958)	Brachychthoniidae	thelytokous	inference
Liochthonius fimbriatissimus Hammer, 1962	Brachychthoniidae	thelytokous	inference
Liochthonius mollis (Hammer, 1958)	Brachychthoniidae	thelytokous	inference
Liochthonius moritzi Balogh & Mahunka, 1983	Brachychthoniidae	thelytokous	inference

Liochthonius neglectus Moritz, 1976	Brachychthoniidae	thelytokous	inference
Liochthonius peduncularius (Strenzke, 1951)	Brachychthoniidae	thelytokous	inference
Liochthonius perfusorius Moritz, 1976	Brachychthoniidae	thelytokous	inference
Liochthonius propinquus Niedbała, 1972	Brachychthoniidae	thelytokous	inference
Liochthonius pseudohystricinus Balogh & Mahunka, 1983	Brachychthoniidae	thelytokous	inference
Liochthonius rigidisetosus Hammer, 1962	Brachychthoniidae	thelytokous	inference
Liochthonius sellnicki (Thor, 1930)	Brachychthoniidae	thelytokous	sex ratio
Liochthonius simplex (Forsslund, 1942)	Brachychthoniidae	thelytokous	inference
Liochthonius strenzkei Forsslund, 1963	Brachychthoniidae	thelytokous	inference
Liochthonius tuxeni (Forsslund, 1957)	Brachychthoniidae	thelytokous	inference
Lohmannia hungarorum Mahunka, 1980	Lohmanniidae	thelytokous	inference
Lyroppia dongnaiensis Ermilov & Anichkin, 2013	Oppiidae	sexual	inference
Machadobelba longiciliata Ermilov, Sandmann, Marian & Maraun, 2013	Machadobelbidae	sexual	inference
Machuella ventrisetosa Hammer, 1961	Machuellidae	sexual	inference
Maculobates breviporosus Mahunka, 1980	Liebstadiidae	sexual	inference
Mahunkana japonica (Aoki & Karasawa, 2007)	Eremulidae	sexual	inference
Malacoangelia remigera Berlese, 1913	Hypochthoniidae	thelytokous	sex ratio
Malaconothrus angulatus Hammer, 1958	Malaconothridae	thelytokous	inference
Malaconothrus dipankari Saha & Sanyal, 1996	Malaconothridae	thelytokous	inference
Malaconothrus dorsofoveolatus Hammer, 1979	Malaconothridae	thelytokous	inference
Malaconothrus geminus (Hammer, 1972)	Malaconothridae	thelytokous	inference
Malaconothrus gracilis Hammen, 1952	Malaconothridae	thelytokous	sex ratio
Malaconothrus monodactylus (Michael, 1888)	Malaconothridae	thelytokous	inference
Masthermannia mammillaris (Berlese, 1904)	Nanhermanniidae	thelytokous	inference
Medioppia media (Mihelčič, 1956)	Oppiidae	sexual	inference
Medioppia obsoleta (Paoli, 1908)	Oppiidae	sexual	inference
Megalotocepheus brevisetus (Mahunka, 1989)	Otocepheidae	sexual	inference
Megalotocepheus crinitus (Berlese, 1905)	Otocepheidae	sexual	inference
Melanozetes mollicomus (Koch, 1839)	Ceratozetidae	sexual	inference
Meristacarus heterotrichus Csiszár, 1961	Lohmanniidae	thelytokous	inference
Meristacarus porcula Grandjean, 1934	Lohmanniidae	thelytokous	inference
Meristacarus sundensis Hammer, 1979	Lohmanniidae	thelytokous	inference
Mesoplophora leviseta Hammer, 1979	Mesoplophoridae	sexual	inference

Mesoplophora michaeliana Berlese, 1904	Mesoplophoridae	sexual	inference
Mesoplophora pulchra Sellnick, 1928	Mesoplophoridae	sexual	inference
Mesotritia nuda (Berlese, 1887)	Oribotritiidae	sexual	inference
Metabelba flagelliseta Bulanova-Zachvatkina, 1965	Damaeidae	sexual	inference
Metabelba papillipes (Nicolet, 1855)	Damaeidae	sexual	inference
Metabelba propexa (Kulczynski, 1902)	Damaeidae	sexual	inference
Metabelba pulverosa Strenzke, 1953	Damaeidae	sexual	inference
Metabelbella interlamellaris Pérez-Íñigo, 1987	Damaeidae	sexual	inference
Micreremus brevipes (Michael, 1888)	Micreremidae	sexual	inference
Microppia minus (Paoli, 1908)	Oppiidae	thelytokous	sex ratio
Microtegeus borhdii Balogh & Mahunka, 1974	Microtegeidae	sexual	inference
Microtegeus reticulatus Aoki, 1965	Microtegeidae	sexual	inference
Microtegeus similis Balogh & Mahunka, 1980	Microtegeidae	sexual	inference
Microtritia minima (Berlese, 1904)	Euphthiracaridae	thelytokous	sex ratio
Microtritia tropica Märkel, 1964	Euphthiracaridae	sexual	inference
Microzetes adansoni (Lions, 1966)	Microzetidae	sexual	inference
Microzetes caucasicus (Krivolutsky, 1967)	Microzetidae	sexual	inference
Microzetes mirandus (Berlese, 1908)	Microzetidae	sexual	inference
Microzetorchestes emeryi (Coggi, 1898)	Zetorchestidae	sexual	inference
Mixacarus foliifer Golosova, 1984	Lohmanniidae	thelytokous	inference
Mochlozetes chambrieri Mahunka, 1990	Mochlozetidae	sexual	inference
Mochlozetes ryukyuensis Aoki, 2006	Mochlozetidae	sexual	inference
Moritzoppia keilbachi (Moritz, 1969)	Oppiidae	sexual	inference
Moritzoppia unicarinata (Paoli, 1908)	Oppiidae	sexual	inference
Mucronothrus nasalis (Willmann, 1929)	Trhypochthoniidae	thelytokous	sex ratio, rearing
Multioppia insularis Mahunka, 1985	Oppiidae	sexual	inference
Multioppia laniseta Moritz, 1966	Oppiidae	sexual	inference
Multioppia tamdao Mahunka, 1988	Oppiidae	sexual	inference
Multioppia wilsoni Aoki, 1964	Oppiidae	sexual	inference
Nanhermannia coronata Berlese, 1913	Nanhermanniidae	thelytokous	sex ratio
Nanhermannia elegantissima Hammer, 1958	Nanhermanniidae	thelytokous	inference
Nanhermannia elegantula Berlese, 1913	Nanhermanniidae	thelytokous	sex ratio, rearing
Nanhermannia nana (Nicolet, 1855)	Nanhermanniidae	thelytokous	sex ratio, rearing

Nehypochthonius porosus Norton & Metz, 1980	Nehypochthoniidae	thelytokous	sex ratio
Neoamerioppia longiclava (Hammer, 1962)	Oppiidae	sexual	inference
Neoamerioppia notata (Hammer, 1958)	Oppiidae	sexual	inference
Neoamerioppia rotunda (Hammer, 1958)	Oppiidae	sexual	inference
Neoamerioppia ventrosquamosa (Hammer, 1979)	Oppiidae	sexual	inference
Neoamerioppia vietnamica (Mahunka, 1988)	Oppiidae	sexual	inference
Neogalumna seniczaki Ermilov and Anichkin, 2010	Galumnidae	sexual	inference
Neogalumna specifica (Ermilov, Sandmann, Klarner, Widyastuti & Scheu, 2015)	Galumnidae	sexual	inference
Neoliochthonius piluliferus (Forsslund, 1942)	Brachychthoniidae	thelytokous	inference
Neoribates aurantiacus (Oudemans, 1914)	Parakalummidae	sexual	inference
Neoribates insignificans (Mahunka, 1995)	Parakalummidae	sexual	inference
Neoribates jacoti (Balogh & Mahunka, 1967)	Parakalummidae	sexual	inference
Neoribates paratuberculatus Ermilov, Shtanchaeva & Subías, 2014	Parakalummidae	sexual	inference
Neoribates spindleformis Ermilov & Anichkink, 2012	Parakalummidae	sexual	inference
Neosuctobelba bituberculata (Ermilov & Anichkin, 2013)	Suctobelbidae	thelytokous	inference
Neosuctobelba transitoria Balogh & Mahunka, 1969	Suctobelbidae	thelytokous	inference
Neotrichoppia confinis (Paoli, 1908)	Oppiidae	thelytokous	inference
Nortonbelba italica Bernini, 1980	Damaeidae	sexual	inference
Nothrus anauniensis Canestrini & Fanzago, 1876	Nothridae	thelytokous	sex ratio, rearing
Nothrus borussicus Sellnick, 1928	Nothridae	thelytokous	sex ratio
Nothrus palustris Koch, 1839	Nothridae	thelytokous	sex ratio, rearing
Nothrus pratensis Sellnick, 1928	Nothridae	thelytokous	inference
Nothrus shapensis Krivolutsky, 1998	Nothridae	thelytokous	inference
Nothrus silvestris Nicolet, 1855	Nothridae	thelytokous	sex ratio, rearing
Nothrus silvicus Jacot, 1937	Nothridae	thelytokous	sex ratio
Nothrus willmanni Mahunka, 1983	Nothridae	thelytokous	inference
Notophthiracarus heterotrichus (Mahunka, 1979)	Phthiracaridae	sexual	inference
Novosuctobelba andrassyi (Balogh & Mahunka, 1981)	Suctobelbidae	thelytokous	inference
Novosuctobelba baculifera (Balogh & Mahunka, 1981)	Suctobelbidae	thelytokous	inference
Novosuctobelba inenodabilis (Hammer, 1979)	Suctobelbidae	sexual	inference
Odontocepheus oblongus (Banks, 1895)	Carabodidae	sexual	inference
Ophidiotrichus tectus (Michael, 1884)	Oribatellidae	sexual	sex ratio

Oppia denticulata (G. & R. Canestrini, 1882)	Oppiidae	sexual	inference
Oppia nitens Koch, 1836	Oppiidae	thelytokous	sex ratio
Oppiella translamellata (Subías, 1980)	Oppiidae	sexual	inference
Oppiella nova (Oudemans, 1902)	Oppiidae	thelytokous	sex ratio, rearing
Oribata ornata Coggi, 1900	Oribatellidae	sexual	inference
Oribatella berlesei (Michael, 1898)	Oribatellidae	sexual	inference
Oribatella calcarata (Koch, 1835)	Oribatellidae	sexual	sex ratio
Oribatella colchica Krivolutsky, 1974	Oribatellidae	sexual	inference
Oribatella foliata Krivolutsky, 1974	Oribatellidae	sexual	inference
Oribatella gerdweigmanni Ermilov & Anichkin, 2012	Oribatellidae	sexual	inference
Oribatella hungarica Balogh, 1943	Oribatellidae	sexual	inference
Oribatella longispina Berlese, 1915	Oribatellidae	sexual	inference
Oribatella malaya Balogh & Mahunka, 1974	Oribatellidae	sexual	inference
Oribatella nigra Kulijev, 1967	Oribatellidae	sexual	inference
Oribatella quadricornuta (Michael, 1880)	Oribatellidae	sexual	sex ratio
Oribatella sexdentata Berlese, 1916	Oribatellidae	sexual	inference
Oribatella superbula (Berlese, 1904)	Oribatellidae	sexual	inference
Oribatella umaetluisorum Ermilov & Anichkin, 2012	Oribatellidae	sexual	inference
Oribatula interrupta (Willmann, 1939)	Oribatulidae	sexual	inference
Oribatula pannonica Willmann, 1949	Oribatulidae	sexual	inference
Oribatula tibialis (Nicolet, 1855)	Oribatulidae	sexual	sex ratio
Oribella pectinata (Michael, 1885)	Oribellidae	sexual	inference
Oribotritia berlesei (Michael, 1898)	Oribotritiidae	sexual	inference
Oromurcia bicuspidata Thor, 1930	Ceratozetidae	sexual	inference
Otocepheus duplicornutus Aoki, 1965	Otocepheidae	sexual	inference
Otocepheus excelsus Aoki, 1965	Otocepheidae	sexual	inference
Otocepheus heterosetiger Aoki, 1965	Otocepheidae	sexual	inference
Otocepheus spatulatus Mahunka, 2000	Otocepheidae	sexual	inference
Otocepheus vietnamicus Ermilov & Anichkin, 2011	Otocepheidae	sexual	inference
Oxyamerus spathulatus Aoki, 1965	Oxyameridae	sexual	inference
Oxyoppia bituberculata (Balogh, 1958)	Oppiidae	sexual	inference
Oxyoppia cubana Balogh & Mahunka, 1980	Oppiidae	sexual	inference

Oxyoppia suramericana (Hammer, 1958)	Oppiidae	sexual	inference
Oxyoppia yepesensis Muñoz-Mingarro, 1987	Oppiidae	sexual	inference
Palaeacarus hystricinus Trägårdh, 1932	Palaeacaridae	thelytokous	sex ratio
Pantelozetes cavaticus (Kunst, 1962)	Oribellidae	sexual	inference
Pantelozetes paolii (Oudemans, 1913)	Oribellidae	sexual	sex ratio
Papillacarus hirsutus (Aoki, 1961)	Lohmanniidae	thelytokous	inference
Papillacarus polygonatus Ermilov & Anichkin, 2011	Lohmanniidae	thelytokous	inference
Papillacarus pseudoaciculatus Mahunka, 1980	Lohmanniidae	thelytokous	inference
Papillacarus ramosus Balogh, 1961	Lohmanniidae	thelytokous	inference
Papillonotus hauseri Mahunka, 1988	Papillonotidae	sexual	inference
Parabelbella meridiana (Norton, 1979)	Damaeidae	sexual	inference
Parachipteria punctata (Nicolet, 1855)	Achipteriidae	sexual	inference
Parasuctobelba compacta (Woas, 1986)	Suctobelbidae	thelytokous	inference
Parasuctobelba complexa (Hammer, 1958)	Suctobelbidae	thelytokous	inference
Parasuctobelba subcomplex a (Balogh & Mahunka, 1968)	Suctobelbidae	thelytokous	inference
Paratritia baloghi Moritz, 1966	Oribotritiidae	sexual	inference
Pasocepheus kirai (Aoki, 1976)	Carabodidae	sexual	inference
Passalozetes africanus Grandjean, 1932	Passalozetidae	sexual	inference
Pedrocortesella vietnamica Ermilov & Anichkin, 2014	Pheroliodidae	sexual	inference
Pelops phaenotus Koch, 1844	Phenopelopoidea	sexual	inference
Peloptulus reticulatus Mihelčič, 1957	Phenopelopidae	sexual	inference
Peloribates europaeus Willmann, 1935	Haplozetidae	sexual	inference
Peloribates glaber Mihelčič, 1956	Haplozetidae	sexual	inference
Peloribates kaszabi Mahunka, 1988	Haplozetidae	sexual	inference
Peloribates rangiroaensi s Hammer, 1972	Haplozetidae	sexual	inference
Peloribates stellatus Balogh & Mahunka, 1967	Haplozetidae	sexual	inference
Pergalumna altera (Oudemans, 1956)	Galumnidae	sexual	inference
Pergalumna annulata Mahunka, 1995	Galumnidae	sexual	inference
Pergalumna cattienica Ermilov & Anichkin, 2011	Galumnidae	sexual	inference
Pergalumna corniculata (Berlese, 1905)	Galumnidae	sexual	inference
Pergalumna formicaria (Berlese, 1995)	Galumnidae	sexual	sex ratio
Pergalumna hauseri Mahunka, 1995	Galumnidae	sexual	inference
Pergalumna hawaiiensis (Jacot, 1934)	Galumnidae	sexual	inference

Pergalumna indistincta Ermilov and Anichkin, 2011	Galumnidae	sexual	inference
Pergalumna kotschyi Mahunka, 1989	Galumnidae	sexual	inference
Pergalumna margaritata Mahunka, 1989	Galumnidae	sexual	inference
Pergalumna nervosa (Berlese, 1915)	Galumnidae	sexual	sex ratio
Pergalumna panayensis Ermilov & Corpuz-Raros, 2015	Galumnidae	sexual	inference
Pergalumna paraindistincta Ermilov, Sandmann, Klarner, Widyastuti & Scheu, 2015	Galumnidae	sexual	inference
Pergalumna petrichosa Mahunka, 1995	Galumnidae	sexual	inference
Pergalumna pseudosejugalis Ermilov and Anichkin, 2012	Galumnidae	sexual	inference
Pergalumna pterinervis (Canestrini, 1898)	Galumnidae	sexual	inference
Pergalumna willmanni (Krivolutskaja, 1952)	Galumnidae	sexual	inference
Perscheloribates subtropicus (Hammer, 1961)	Scheloribatidae	sexual	inference
Perxylobates crassisetosus Ermilov & Anichkin, 2011	Protoribatidae	sexual	inference
Perxylobates thanhoaensis Ermilov, Vu, Trinh & Dao, 2011	Protoribatidae	sexual	inference
Phauloppia lucorum (Koch, 1841)	Oribatulidae	sexual	inference
Phauloppia nemoralis (Berlese, 1916)	Oribatulidae	sexual	inference
Pheroliodes intermedius (Hammer, 1961)	Pheroliodidae	sexual	inference
Phthiracarus bryobius Jacot, 1930	Phthiracaridae	sexual	inference
Phthiracarus crassus Niedbala, 1983	Phthiracaridae	sexual	inference
Phthiracarus ferrugienus (Koch, 1841)	Phthiracaridae	sexual	inference
Phthiracarus globosus (Koch, 1841)	Phthiracaridae	sexual	inference
Phthiracarus italicus (Oudemans, 1900)	Phthiracaridae	sexual	inference
Phthiracarus laevigatus (Koch, 1841)	Phthiracaridae	sexual	inference
Phthiracarus lentulus (Koch, 1841)	Phthiracaridae	sexual	inference
Phthiracarus longulus (Koch, 1841)	Phthiracaridae	sexual	inference
Phyllozetes emmae (Berlese, 1910)	Cosmochthoniidae	sexual	inference
Pilobates carpetanus Pérez-Íñigo, 1969	Haplozetidae	sexual	inference
Pilogalumna crassiclava (Berlese, 1915)	Galumnidae	sexual	inference
Pilogalumna ornatula Grandjean, 1956	Galumnidae	sexual	inference
Pilogalumna tenuiclavata (Berlese, 1908)	Galumnidae	sexual	inference
Plasmobates asiaticus Aoki, 1973	Plasmobatidae	sexual	inference
Platyliodes doderleini (Berlese, 1883)	Neoliodidae	sexual	inference
Platynothrus peltifer (Koch, 1839)	Crotoniidae	thelytokous	sex ratio, rearing
Platynothrus punctatus (L. Koch, 1879)	Crotoniidae	thelytokous	sex ratio

Plenotocepheus neotropicus Ermilov, Sandmann, Marian & Maraun, 2013	Tetracondylidae	sexual	inference
Podopterotegaeus tectus Aoki, 1969	Podopterotegaeidae	thelytokous	sex ratio
Poecilochthonius italicus (Berlese, 1910)	Brachychthoniidae	thelytokous	inference
Poecilochthonius spiciger (Berlese, 1910)	Brachychthoniidae	thelytokous	sex ratio
Porobelba spinosa (Sellnick, 1920)	Damaeidae	sexual	inference
Poroliodes farinosus (Koch, 1839)	Neoliodidae	sexual	inference
Protoribates bisculpturatus (Mahunka, 1988)	Protoribatidae	sexual	inference
Protoribates capucinus Berlese, 1908	Protoribatidae	thelytokous	inference
Protoribates cattienensis Ermilov & Anichkin, 2011	Protoribatidae	sexual	inference
Protoribates heterodactylus Ermilov & Anichkin, 2011	Protoribatidae	sexual	inference
Protoribates lophotrichus (Berlese, 1904)	Protoribatidae	thelytokous	inference
Protoribates maximus (Mahunka, 1988)	Protoribatidae	sexual	inference
Protoribates paracapucinus (Mahunka, 1988)	Protoribatidae	thelytokous	inference
Pseuderemulus gladiator Balogh & Mahunka, 1968	Eremulidae	sexual	inference
Pseudoamerioppia barrancensis (Hammer, 1961)	Oppiidae	sexual	inference
Pseudotocepheus setiger (Hammer, 1972)	Tetracondylidae	sexual	inference
Pterochthonius angelus (Berlese, 1910)	Atopochthoniidae	thelytokous	sex ratio
Pulchroppia elegans Hammer, 1979	Oppiidae	sexual	inference
Pulchroppia roynortoni Ermilov & Anichkin, 2011	Oppiidae	sexual	inference
Punctoribates punctum (Koch, 1839)	Punctoribatidae	sexual	sex ratio
Punctoribates sellnicki Willmann, 1928	Punctoribatidae	sexual	inference
Quadroppia circumita (Hammer, 1961)	Quadroppiidae	sexual	sex ratio
Quadroppia media Gordeeva, 1983	Quadroppiidae	sexual	inference
Quadroppia monstruosa Hammer, 1979	Quadroppiidae	thelytokous	inference
Quadroppia quadricarinata (Michael, 1885)	Quadroppiidae	thelytokous	sex ratio
Ramusella chulumaniensis (Hammer, 1958)	Oppiidae	sexual	inference
Ramusella clavipectinata (Michael, 1885)	Oppiidae	sexual	inference
Ramusella elliptica (Berlese, 1908)	Oppiidae	sexual	inference
Ramusella furcata (Willmann, 1928)	Oppiidae	sexual	inference
Ramusella insculpta (Paoli, 1908)	Oppiidae	sexual	inference
Ramusella mihelcici (Pérez-Íñigo, 1965)	Oppiidae	sexual	inference
Ramusella terricola Subías & Rodríguez, 1986	Oppiidae	sexual	inference
Rastellobata rastelligera (Berlese, 1908)	Amerobelbidae	sexual	inference

Rhinoppia subpectinata (Oudemans, 1900)	Oppiidae	sexual	sex ratio
Rhynchoribates mirus Beck, 1961	Rhynchoribatidae	sexual	inference
Rostrozetes carinatus Beck, 1965	Haplozetidae	thelytokous	inference
Rostrozetes florens (Balogh, 1970)	Haplozetidae	sexual	inference
Rostrozetes ovulum (Berlese, 1908)	Haplozetidae	thelytokous	sex ratio
Rostrozetes shibai Aoiki, 1976	Haplozetidae	sexual	inference
Sabahtritia sarawak Mahunka, 1996	Synichotritiidae	sexual	inference
Sadocepheus undulatus Aoki, 1965	Cepheidae	sexual	inference
Scapheremaeus bicornutus Hammer, 1971	Cymbaeremaeidae	sexual	inference
Scapheremaeus corniger (Berlese, 1908)	Cymbaeremaeidae	sexual	inference
Scapheremaeus foveolatus Mahunka, 1987	Cymbaeremaeidae	sexual	inference
Schalleria brevisetosa Ermilov, Sandmann & Maraun, 2013	Microzetidae	sexual	inference
Schalleria pectinata Ermilov, Sandmann & Maraun, 2013	Microzetidae	sexual	inference
Scheloribates ascendens Weigmann & Wunderle, 1990	Scheloribatidae	sexual	inference
Scheloribates barbatulus Mihelčič, 1956	Scheloribatidae	sexual	inference
Scheloribates elegans Hammer, 1958	Scheloribatidae	sexual	inference
Scheloribates elegantulus Hammer, 1961	Scheloribatidae	sexual	inference
Scheloribates fimbriatus Thor, 1930	Scheloribatidae	sexual	inference
Scheloribates kraepelini (Berlese, 1908)	Scheloribatidae	sexual	inference
Scheloribates laevigatus (Koch, 1835)	Scheloribatidae	sexual	sex ratio
Scheloribates lanceoliger (Berlese, 1908)	Scheloribatidae	sexual	sex ratio
Scheloribates laticlava Hammer, 1961	Scheloribatidae	sexual	inference
Scheloribates luminosus Hammer, 1961	Scheloribatidae	sexual	inference
Scheloribates luteomarginatus Hammer, 1958	Scheloribatidae	sexual	inference
Scheloribates mahunkai Subias, 2010	Scheloribatidae	sexual	inference
Scheloribates pallidulus (Koch, 1841)	Scheloribatidae	sexual	inference
Scheloribates vulgaris Hammer, 1961	Scheloribatidae	sexual	inference
Scutovertex sculptus Michael, 1879	Scutoverticidae	sexual	inference
Sellnickochthonius cricoides (Weis-Fogh, 1948)	Brachychthoniidae	thelytokous	sex ratio
Sellnickochthonius elsosneadensis (Hammer, 1958)	Brachychthoniidae	thelytokous	inference
Sellnickochthonius foliatus (Hammer, 1958)	Brachychthoniidae	thelytokous	inference
Sellnickochthonius honestus (Moritz, 1976)	Brachychthoniidae	thelytokous	inference
Sellnickochthonius immaculatus (Forsslund, 1942)	Brachychthoniidae	thelytokous	inference

Sellnickochthonius lydiae (Jacot, 1938)	Brachychthoniidae	thelytokous	sex ratio
Sellnickochthonius muara Mahunka, 1995	Brachychthoniidae	thelytokous	inference
Sellnickochthonius zelawaiensis (Sellnick, 1928)	Brachychthoniidae	thelytokous	inference
Siculobata leontonycha (Berlese, 1910)	Hemileiidae	sexual	inference
Singabodes rarus Mahunka, 1998	Carabodidae	sexual	inference
Solenozetes carinatus (Hammer, 1961)	Plasmobatidae	sexual	inference
Solenozetes flagellifer Mahunka, 1983	Plasmobatidae	sexual	inference
Sphaerochthonius splendidus (Berlese, 1904)	Sphaerochthoniidae	sexual	inference
Sphaerozetes chavinensis (Hammer, 1961)	Ceratozetidae	sexual	inference
Sphaerozetes piriformis (Nicolet, 1855)	Ceratozetidae	sexual	inference
Steganacarus applicatus (Sellnick, 1920)	Phthiracaridae	sexual	inference
Steganacarus brevipilus (Berlese, 1923)	Phthiracaridae	sexual	inference
Steganacarus carinatus (Koch, 1841)	Phthiracaridae	sexual	inference
Steganacarus herculeanus Willmann, 1953	Phthiracaridae	sexual	inference
Steganacarus magnus (Nicolet, 1855)	Phthiracaridae	sexual	inference
Steganacarus patruelis Niedbala, 1983	Phthiracaridae	sexual	inference
Steganacarus spinosus (Sellnick, 1920)	Phthiracaridae	sexual	inference
Sternoppia brasiliensis Franklin & Woas, 1992	Oppiidae	sexual	inference
Sternoppia incisa Balogh & Mahunka, 1977	Oppiidae	sexual	inference
Sternoppia mirabilis Balogh & Mahunka, 1968	Oppiidae	sexual	inference
Sternoppia paraincisa Ermilov, Sandmann, Marian & Maraun, 2013	Oppiidae	sexual	inference
Striatoppia lanceolata Hammer, 1972	Oppiidae	sexual	inference
Striatoppia modesta Mahunka, 1988	Oppiidae	sexual	inference
Striatoppia opuntiseta Balogh & Mahunka, 1968	Oppiidae	sexual	inference
Subiasella quadrimaculata (Evans, 1952)	Oppiidae	sexual	inference
Suctobelba altvateri Moritz, 1970	Suctobelbidae	thelytokous	inference
Suctobelba granulata Hammen, 1952	Suctobelbidae	thelytokous	inference
Suctobelbata prelli (Märkel & Meyer, 1958)	Suctobelbidae	thelytokous	inference
Suctobelbella acutidens duplex (Strenzke, 1950)	Suctobelbidae	thelytokous	inference
Suctobelbella acutidens sarekensis (Forsslund, 1941)	Suctobelbidae	thelytokous	inference
Suctobelbella acutidens (Forsslund, 1941)	Suctobelbidae	thelytokous	inference

Suctobelbella carcharodon Moritz, 1966	Suctobelbidae	thelytokous	inference
Suctobelbella elegantula (Hammer, 1958)	Suctobelbidae	thelytokous	inference
Suctobelbella forsslundi (Strenzke, 1950)	Suctobelbidae	thelytokous	inference
Suctobelbella hurshi Jacot, 1937	Suctobelbidae	thelytokous	sex ratio
Suctobelbella indica (Haq, 1978)	Suctobelbidae	thelytokous	inference
Suctobelbella laevis Jacot, 1938	Suctobelbidae	thelytokous	sex ratio
Suctobelbella latirostris (Strenzke, 1950)	Suctobelbidae	thelytokous	inference
Suctobelbella longicuspis Jacot, 1937	Suctobelbidae	thelytokous	inference
Suctobelbella palustris (Forsslund, 1953)	Suctobelbidae	thelytokous	inference
Suctobelbella parallelodentata Hammer, 1979	Suctobelbidae	thelytokous	inference
Suctobelbella peracuta (Balogh & Mahunka, 1980)	Suctobelbidae	thelytokous	inference
Suctobelbella phylliformis Ermilov, Shtanchaeva & Subías, 2014	Suctobelbidae	thelytokous	inference
Suctobelbella pilifera (Mahunka, 1978)	Suctobelbidae	thelytokous	inference
Suctobelbella plumata (Hammer, 1979)	Suctobelbidae	thelytokous	inference
Suctobelbella reticulata (Hammer, 1982)	Suctobelbidae	thelytokous	inference
Suctobelbella semiplumosa (Balogh & Mahunka, 1967)	Suctobelbidae	thelytokous	inference
Suctobelbella sicilifera (Hammer, 1961)	Suctobelbidae	thelytokous	inference
Suctobelbella similis (Forsslund, 1941)	Suctobelbidae	thelytokous	sex ratio
Suctobelbella subcornigera (Forsslund, 1941)	Suctobelbidae	thelytokous	sex ratio
Suctobelbella variosetosa (Hammer, 1961)	Suctobelbidae	thelytokous	inference
Suctobelbila approximata Balogh, 1968	Suctobelbidae	thelytokous	inference
Suctobelbila dentata (Hammer, 1961)	Suctobelbidae	thelytokous	inference
Suctobelbila multituberculata Hammer, 1979	Suctobelbidae	thelytokous	inference
Suctobelbila peruensis Woas, 1986	Suctobelbidae	thelytokous	inference
Suctobelbila tuberculata Aoki, 1970	Suctobelbidae	thelytokous	inference
Suctobelbila undulata Hammer, 1979	Suctobelbidae	thelytokous	inference
Suctoribates foliatus Mahunka, 1997	Rhynchoribatidae	sexual	inference
Suctotegeus tumescitus Mahunka, 1987	Microtegeidae	sexual	inference
Sumatrotritia inusitata Mahunka, 1989	Euphthiracaridae	sexual	inference
Synchthonius crenulatus (Jacot, 1938)	Brachychthoniidae	thelytokous	inference
Taiwanoppia hungarorum (Mahunka, 1988)	Oppiidae	sexual	inference
Tecteremaeus cornutus Hammer, 1961	Arceremaeidae	sexual	inference
Tecteremaeus hauseri Mahunka, 1982	Arceremaeidae	sexual	inference

Tecteremaeus incompletus Mahunka, 1988	Arceremaeidae	sexual	inference
Tectocepheus alatus Berlese, 1913	Tectocepheidae	thelytokous	inference
Tectocepheus minor Berlese, 1903	Tectocepheidae	thelytokous	sex ratio
Tectocepheus sarekensis (Trägårdh, 1910)	Tectocepheidae	thelytokous	sex ratio
Tectocepheus velatus (Michael, 1880)	Tectocepheidae	thelytokous	sex ratio
Tegeocranellus laevis (Berlese, 1905)	Tegeocranellidae	sexual	inference
Tegeozetes tunicatus Berlese, 1913	Tectocepheidae	sexual	inference
Teratoppia pluripectinata Balogh & Mahunka, 1978	Oppiidae	sexual	inference
Thamnacarus longisetosus Bulanova-Zachvatkina, 1978	Lohmanniidae	thelytokous	inference
Totobates discifer Hammer, 1961	Liebstadiidae	sexual	inference
Trachyoribates ampulla (Berlese, 1905)	Haplozetidae	sexual	inference
Trhypochthoniellus longisetus (Berlese, 1904)	Trhypochthoniidae	thelytokous	inference
Trhypochthonius americanus (Ewing, 1908)	Trhypochthoniidae	thelytokous	sex ratio, rearing
Trhypochthonius tectorum (Berlese, 1896)	Trhypochthoniidae	thelytokous	sex ratio, rearing
Trichogalumna nipponica (Aoki, 1966)	Galumnidae	sexual	inference
Trichoribates caucasicus Shaldybina, 1971	Ceratozetidae	sexual	inference
Trichoribates hammerae Subías, 2010	Ceratozetidae	sexual	inference
Trichoribates incisellus (Kramer, 1897)	Ceratozetidae	sexual	inference
Trichoribates novus (Sellnick, 1928)	Ceratozetidae	sexual	inference
Tritegeus bifidatus Grandjean, 1953	Cepheidae	sexual	inference
Trypogalumnella poronota Mahunka, 1995	Galumnellidae	sexual	inference
Tyrphonothrus cordisetus (Mahunka, 1993)	Malaconothridae	thelytokous	inference
Tyrphonothrus maior Berlese, 1910	Malaconothridae	thelytokous	inference
Tyrphonothrus saxosus (Knülle, 1957)	Malaconothridae	thelytokous	inference
Unguizetes cattienensis Ermilov & Anichkin, 2013	Mochlozetidae	sexual	inference
Unguizetes clavatus Aoki, 1967	Mochlozetidae	sexual	inference
Unguizetes latus Ermilov & Anichkin, 2013	Mochlozetidae	sexual	inference
Unguizetes sphaerula (Berlese, 1905)	Mochlozetidae	sexual	inference
Wallworkoppia machadoi (Balogh, 1958)	Oppiidae	sexual	inference
Xenillus clypeator Robineau-Desvoidy, 1839	Xenillidae	sexual	inference
Xenillus tegeocranus (Hermann, 1804)	Xenillidae	sexual	inference
Xenolohmannia comosa Balogh, 1984	Lohmanniidae	thelytokous	inference

Yoshiobodes aokii Mahunka, 1987	Carabodidae	sexual	inference
Yoshiobodes biconcavus Ermilov, Shtanchaeva, Subías & Anichkin, 2014	Carabodidae	sexual	inference
Yoshiobodes nakatamarii (Aoki, 1973)	Carabodidae	sexual	inference
Zachvatkinibates quadrivertex (Halbert. 1920)	Punctoribatidae	sexual	inference
Zetomimus furcatus Warburton and Pearce, 1905	Ceratozetidae	sexual	inference
Zetorchestes grandjeani Krisper, 1987	Zetorchestidae	sexual	inference
Zetorchestes novaguineanus Krisper, 1987	Zetorchestidae	sexual	inference
Zetorchestes saltator Oudemans, 1915	Zetorchestidae	sexual	inference

Beck L (1969) Zum jahreszeitlichen Massenwechsel zweier Oribatidenarten (Acari) im neotropischen Überschwemmungswald. Zool. Anz. (Suppl.) 32, 535-540

Cianciolo JM (2001) Ecological distribution of asexual oribatid mites: a test of the Red Queen hypothesis. MSc Thesis, State University of New York, Syracuse, New Y[illegible]

Domes K, Scheu S, Maraun M (2007) Resources and sex: soil re-colonization by sexual and parthenogenetic oribatid mites. Pedobiologia 51, 1-11

Fujikawa T (1988) Biological features of *Oppiella nova* (Oudemans) in a Nature farming field. Edaphologia 38, 1-10

Grandjean F (1941) Statistique sexuelle et parthénogenèse chez les Oribates (Acariens). C. R. Séanc. Ac. Sci. 212, 463-467

Grandjean F (1947) Observations sur les Acariens (10e série). Bull. Mus. Nat. Hist. Natur. 19, 76-83

Grandjean F (1950) Étude sur les Lohmanniidae (Oribates, Acariens). Arch. Zool. Exp. Gén. 87, 95-161

Grandjean F (1955) Observations sur les Oribates (32e série). Bull. Mus. Nat. Hist. Natur. 27, 212-219

Grandjean F (1961) Nouvelles observations sur les Oribates (Ire série). Acarologia 3, 206-231

Harding DJL (1969) Seasonal changes in the abundance of Cryptostigmata in the forest floor of an oak woodland, pp. 31-35. In G. O. Evans (ed.), Proc. 2nd Int. Congr[illegible]

Kaneko N (1989) Life histories of four oribatid mite species in a mull type soil in a cool temperate forest in Japan. Pedobiologia 33, 117-126

Lions JC (1967) La prelarve de *Rhysotritia ardua* (C. L. Koch) 1836 (Acarien, Oribate). Acarologia 9, 273-83

Luxton, M (1981) Studies on the oribatid mites of a Danish beech wood soil. IV. Developmental biology. Pedobiologia 21, 312-340

Marshall VG, Reeves RM (1970) *Trichthonius majestus* , a new species of oribatid mite (Acariña: Cosmochthoniidae) from North America. Acarologia 12, 623-632

Norton RA, Metz L (1980) Nehypochthoniidae (Acari: Oribatei), a new mite family from the southeastern United States. Ann. Entomol. Soc. Amer. 73, 54-62

Norton RA, Kethley JB, Johnston DE, OConnor BM (1993) Phylogenetic perspectives on genetic systems and reproductive modes of mites. In: Wrensch D, Ebbert M (

Palmer SC, Norton RA (1990) Further experimental proof of thelytokous parthenogenesis in oribatid mites (Acari: Oribatida: Desmonomata). Exper. Appl. Acarol. 8,

Palmer SC, Norton RA (1991) Taxonomic, geographic, and seasonal distribution of thelytokous parthenogenesis in Desmonomata (Acari: Oribatida). Exper. Appl. Aca

Ryabinin NA, Pankov AN (1987) The role of parthenogenesis in the biology of armored mites. Ecologia, Academia Nauka, USSR 1987, pp. 62-64

Saichuae P, Gerson U, Henis Y (1972) Observations on the feeding and life history of the mite *Nothrus biciliatus* (Koch). Soil Biol. Biochem. 4, 155-164

Sengbusch HG, Sengbusch CH (1970) Post-embryonic development of *Oppia nitens* (Acarina: Oribatei). J. N.Y. Entomol. Soc. 78, 207-214

Sitnikova LG (1962) A brief outline of the oribatid mite fauna (Acariformes, Oribatei) of the Leningrad region. Trud. Zool. lnstit. Acad. Nauk, CCCP 31, 429-452 (In Rus

Solhoy T (1975) Dynamics of Oribatei populations on Hardangervidda, pp. 111-116. In F. Wielgolaski (ed.). Fennoscandian Tundra Ecosystems. Part 2: Animals and Sy

Subias LS (2022) Listado sistemático, sinonímico y biogeográfico de los ácaros oribátidos (Acariformes: Oribatida) del mundo (excepto fósiles) (17ª actualización). ht

Trave J (1963) Ecologie et biologie des Oribates (Acariens) saxicoles et arboricoles. Vie et Milieu, Suppl. 14, pp. i-viii and 1-267

Wehner K, Schuster R, Simons NK, Norton RA, Blüthgen N, Heethoff M (2021) How land-use intensity affects sexual and parthenogenetic oribatid mites in temperate

n and area (km²) of the studied oribatid mite species.

Reference
Domes et al. (2007)
Wehner et al. (2021)
Grandjean (1941), Lions (1967)
Harding (1969), Wehner et al. (2021)
Wehner et al. (2021)
Palmer & Norton (1990, 1991)
Palmer & Norton (1990, 1991)
Cianciolo (2001)
Palmer & Norton (1990, 1991)

Kaneko (1989)
Cianciolo (2001)
Domes et al. (2007)
Wehner et al. (2021)
Wehner et al. (2021)
Cianciolo (2001)
Wehner et al. (2021)

Palmer & Norton (1991)
Travé (1963)
Grandjean (1941), Palmer & Norton (1991)
Taberly (1987), Palmer & Norton (1991)
Wehner et al. (2021)
Wehner et al. (2021)

Norton et al. (1993)
Luxton (1981), Cianciolo (2001), Wehner et al. (2021)
Wehner et al. (2021)
Wehner et al. (2021)
Wehner et al. (2021)
Grandjean (1950)
Cianciolo (2001), Wehner et al. (2021)
Cianciolo (2001)

Grandjean (1955, 1961)
Wehner et al. (2021)
Wehner et al. (2021)
Wehner et al. (2021)

Grandjean 1941
Cianciolo (2001)
Grandjean (1941)
Wehner et al. (2021)
Domes et al. (2007)
Wehner et al. (2021)

Cianciolo (2001)
Wehner et al. (2021)
Norton et al. (1993)
Marshall & Reeves (1970)
Cianciolo (2001)

Palmer & Norton (1990, 1991)
Grandjean (1941), Palmer & Norton (1991)
Wehner et al. (2021)
Grandjean (1941)
Grandjean (1941)
Grandjean (1941), Wehner et al. (2021)
Cianciolo (2001)

Wehner et al. (2021)
Wehner et al. (2021)
Wehner et al. (2021)

Ryabin & Pankov 1987
Norton et al. (1993)
Sitnikova (1962), Palmer & Norton (1991)

Luxton (1981), Wehner et al. (2021)
Grandjean (1941)
Palmer & Norton (1990, 1991)
Domes et al. (2007)
Palmer & Norton (1990, 1991)
Grandjean (1941, 1947)

Norton & Metz (1980)
Saichuae et al. (1972), Palmer & Norton (1991)
Palmer & Norton (1991)
Grandjean (1941), Palmer & Norton (1991)
Grandjean (1941), Luxton (1981), Palmer & Norton (1991)
Palmer & Norton (1991)
Domes et al. (2007)

Sengbusch & Sengbusch (1970)
Luxton (1981), Kaneko (1989)
Wehner et al. (2021)
Domes et al. (2007)
Cianciolo (2001), Wehner et al. (2021)

Grandjean (1954)
Wehner et al. (2021)
Cianciolo (2001)

Wehner et al. (2021)
Grandjean (1941), Taberly (1987)
Palmer & Norton (1991)

Cianciolo (2001)
Wehner et al. (2021)
Norton et al. (1993)
Cianciolo (2001), Wehner et al. (2021)
Cianciolo (2001)
Luxton (1981), Wehner et al. (2021)

Wehner et al. (2021)
Beck (1969)
Cianciolo (2001), Wehner et al. (2021)
Cianciolo (2001)
Wehner et al. (2021)

Norton et al. (1993)

Cianciolo (2001)
Cianciolo (2001)
Cianciolo (2001)
Domes et al. (2007)

Grandjean (1941)
Grandjean (1941)
Grandjean (1941), Solhoy (1975), Fujikawa (1988)
Palmer & Norton (1990, 1991)
Taberly (1951, 1987), Grandjean 1941

ork

[illegible]. Acarology. Akademiai Kiado, Budapest

;eds) Evolution and diversity of sex ratios in insects and mites

149-159

ırol. 12, 67-81

ssian)

ystems Analysis. Berlin: Springer-Verlag

tp://bba.bioucm.es/cont/docs/RO_1.pdf

forests and grasslands in Germany. Exp. Appl. Acarol. 83, 34[illegible]

Distribution (according to Subias, 2022)	Area (km²)
Holártica (Paleártica, y Neártica septentrional), India (Sikkim) e I. Santa	65,811,786
Suiza	41,285
Holártica (Paleártica, y U.S.A.: Virginia)	54,210,785
Europa y Neotropical (Brasil y Argentina)	21,274,830
Semicosmopolita: Holártica, Etiópica, Oriental (India: Kerala y China suroriental) y Nueva Zelanda	101,240,508
Cosmopolita	148,940,000
Paleártica y Oriental (Vietnam y Nepal)	54,478,728
Semicosmopolita (Holártica, Etiópica: Somalia, Oriental y Neotropical: Panamá)	85,213,074
Holártica (Paleártica occidental, Oeste de Siberia y U.S.A.: Minesota)	24,308,220
Paleártica (Paleártica occidental: excepto Norte, y Paquistán)	12,818,317
Europa centromeridional	294,507
Oriental	7,500,000
Pantropical	27,604,311
Sumatra	473,481
Vietnam	331,212
Sumatra	473,481
Tropical (Etiópica: Ghana, Oriental y Norte de Neotropical) y Japón	22,477,975
Holártica y Noreste de Oriental	80,099,270
Este de U.S.A.	1,791,708
Oriental y Neotropical (Perú)	8,779,996
Neotropical	19,000,000
Paleártica meridional (Europa centromeridional e Irán)	1,826,102
Europa centromeridional e Irán	1,826,102
Mediterránea	2,085,292
Ecuador	283,561
Semicosmopolita (Holártica, Etiópica y Neotropical), Sahara occidental	118,336,000
Península Malaya	242,364
Pantropical (excepto Etiópica)	26,500,000

Semicosmopolita: Holártica (Neártica, Paleártica oriental meridional y Cáucaso), Oriental, Neotropical (Centroamérica) y Etiópica (Madagascar)	37,914,527
Norte de Neotropical	9,054
Oriental	7,500,000
Neotropical y África occidental	25,409,409
Oriental	7,500,000
Europa centromeridional y Vietnam	625,719
Semicosmopolita (Holártica, Oriental, Centroamérica y Australiana)	92,721,876
Mediterránea	2,085,292
Paleártica meridional (Mediterránea y Asia centrooccidental)	3,419,776
Paleártica	54,100,000
Holártica (Paleártica, y Neártica septentrional)	65,804,690
Holártica (Paleártica y Canadá)	63,193,507
Holártica, U.S.A. (Illinois) y Este de Rusia asiática	77,000,000
Japón y Oriental (Taiwán y Vietnam)	745,384
Pantropical y Japón	48,977,975
Cuba	10,982
Paleártica occidental (excepto Norte)	12,022,222
Paleártica meridional (Mediterránea y Paleártica oriental meridional)	8,096,403
Neotropical, India (Kerala) e Irán	20,570,458
Pantropical y subtropical	81,935,554
Paleártica, Madagascar y Vietnam	55,012,752
Europa centromeridional	294,507
Paleártica occidental y Asia centrooccidental	22,917,534
Palaearctica meridonal	18,033,333
Neotropical, Norte de India y Japón	20,798,975
Neotropical	19,000,000
Holártica	77,000,000
Paleártica meridional (Europa centromeridional, Irán y Mongolia)	3,392,102
Paleártica	54,100,000
Holártica (Paleártica occidental, Canadá)	77,000,000
Oriental y Australiana (Nueva Guinea	8,286,000
Mediterránea occidental	1,390,195

Italia (Sicilia)	25,426
Paleártica occidental y Méjico	23,555,599
Holártica y China suroriental	78,837,108
Neotropical, Etiópica (Sudáfrica y Etiopía) y subantártica (I. Tristán da Cunha)	21,214,650
Holártica, Norte de Oriental, Etiópica (Etiopía) y Norte de Neotropical	82,975,556
Holártica, Norte de Oriental, Etiópica (Etiopía) y Norte de Neotropical	82,975,556
Neotropical (Perú y Chile)	2,023,808
Boreoalpina	78,249,340
Semicosmopolita (Holártica, Oriental, Neotropical y Antártica)	117,500,000
Holártica y Oriental (China suroriental y Nepal)	78,984,624
Semicosmopolita (Holártica, Norte de Oriental y Neotropical: Brasil y Panamá)	89,503,713
Holártica (Paleártica, y U.S.A.: Virginia) y Centroamérica	54,732,661
Mediterránea	2,085,292
Paleártica meridional (Mediterránea e Irán)	3,616,887
I. Azores	2,351
Holártica	77,000,000
Paleártica occidental y U.S.A. (Virginia)	21,693,835
Paleártica	27,594,161
Holártica y Méjico.	78,972,550
Paleártica	18,822,265
Paleártica (excepto Este de Paleártica oriental)	12,022,222
Paleártica occidental	21,583,050
Holártica (Paleártica y Neártica septentrional)	6,580,469
Polinesia (I. Samoa) y Vietnam	334,054
Paleártica	28,213,333
Holártica (Paleártica occidental, Irán y Neártica septentrional)	34,819,335
Vietnam	331,212
Borneo	751,936
Neotropical	19,000,000
Holártica (Paleártica, y U.S.A.: Carolina del Norte) y China suroriental	56,076,497
Paleártica: Europa y Este de Rusia asiática	13,280,000
Holártica y Oriental (China suroriental y Nepal)	78,984,623
Holártica, Norte de Oriental y Centroamérica	81,592,031

Holártica (Paleártica y Neártica septentrional) y China suroriental	67,641,797
Holártica (Europa, Este de Paleártica oriental y Neártica septentrional)	27,895,801
Boreal	66,823,100
Bolivia	1083301
Neártica	22,900,000
Cosmopolita: Holártica, I. Santa Helena, Oriental, Nueva Zelanda, Neotropical y Antártica	103,769,360
Semicosmopolita: Holártica, Oriental y Australiana (Nueva Zelanda)	84,764,537
Neártica occidental	7,633,333
Holártica (Paleártica occidental, Este de Rusia asiática y Canadá)	33,776,557
Holártica: Paleártica (excepto Este) y Groenlandia	17,432,753
Holártica e I. Seychelles	54,100,455
Paleártica	54,100,000
Paleártica (Paleártica occidental, Irán y Este de Rusia asiática)	26,517,528
Paleártica (Europa, y Paleártica oriental: excepto Este)	30,330,851
Vietnam	331,212
Paraguay	406,752
Cosmopolita (Holártica, Etiópica, Oriental y Neotropical)	125,600,000
Holártica meridional, Norte de Neotropical y China suroriental, Neotropical, U.S.A. (Nuevo Méjico), e India (Tripura)	45,089,634
Australianooriental: Micronesia (I. Marianas) y Vietnam	331,689
Neotropical (Cuba y Ecuador)	393,381
Paleártica meridional (Mediterránea y Asia centrooccidental) y Etiópica	25,519,776
Tropical: Etiópica (Nigeria), Oriental (Java) y Neotropical, y Subtropical: Paleártica meridional y Neártica (U.S.A.: Tejas)	38,779,422
Borneo	751,936
Pantropical y subtropical	81,935,554
Alemania y Mongolia	1,914,672
Holártica y Java	77,126,700
Holártica (Neártica oriental y Europa meridional)	8,950,377
Holártica (Europa, Este de Rusia asiática y U.S.A. oriental)	14,927,422
Paleártica (Italia y Paleártica oriental), Oriental y Australiana (Australia y Nueva Zelanada)	33,534,671
Neotropical	19,000,000
Japón y Oriental (Taiwán y Vietnam)	745,384
Paleártica e India (Sikkim)	54,107,096
Neotropical	19,000,000

Congo, Mediterránea oriental y Java	3,166,655
Oriental y Brasil	15,858,140
Paleártica (Paleártica occidental y Este de Rusia asiática).	24,683,049
Paleártica meridional (Mediterránea y Asia centrooccidental)	3,419,776
U.S.A. oriental	1,647,422
Paleártica	54,100,000
Paleártica occidental	21,583,049
Paleártica occidental	21,583,049
Europa y Mongolia	11,746,000
China suroriental	1,837,107
Neotropical (Chile y Ecuador)	1,027,373
Paleártica occidental y Etiópica: Tanzania e I. Santa Helena	22,528,259
Paleártica	54,100,000
Europa	10,180,000
Australiana (Nueva Guinea) y Oriental (Filipinas y Vietnam)	1,415,382
Holártica	77,000,000
Boreoalpina	78,249,340
Paleártica y tropical: Vietnam y Panama	54,555,071
Holártica y África oriental	56,030,512
Vietnam	331,212
Vietnam	331,212
Vietnam	331,212
Vietnam	331,212
Europa centromeridional	294,597
Vietnam	331,212
Sumatra	473,481
Península Malaya	242,364
Java	1,267
Neotropical	19,000,000
Boreoalpina: Euroatlántica, Cáucaso y Groenlandia	2,951,293
Holártica	77,000,000
Oriental (Java y Borneo)	878,636
Norte de Neotropical + Ecuador	1,188,961

Ecuador	283,561
Vietnam	331,212
Cosmopolita (Paleártica, Neártica, Etiópica: Chad, Oriental, Australiana: Hawái, y Neotropical)	104,775,825
Australia, Pantropical (excepto Etiópica) y Paleártica oriental meridional	32,290,167
Java	126,700
Holártica y China suroriental	78,837,107
Holártica, Oriental y Neotropical	103,500,000
Pantropical (y subtropical): Oriental, Japón, Australiana: I. Tonga, Neotropical: Brasil, y Etiópica: Congo y Madagascar	19,163,263
Oriental	7,500,000
Nueva Guinea y Vietnam	1,117,212
Borneo	751,936
Holártica (Neártica oriental y Cáucaso) y Méjico	10,005,883
Europa centromeridional	294,507
Bali	5,780
Java	126,700
India (Tripura) y Neotropical	19,010,492
Paleártica centromeridional	6,011,111
Holártica y China suroriental	78,837,108
Semicosmopolita: Paleártica, Norte de Oriental, Etiópica (Sudáfrica y Etiopía) y Argentina	63,121,316
Mediterránea oriental + Canarias	702,589
Holártica (Europa centromeridional, Paquistán y Canadá)	10,184,109
Paleártica	54,100,000
Holártica (Paleártica, y U.S.A.: Alaska y Virginia)	55,928,639
Europa centromeridional	294,507
Paleártica, y Etiopía	55,100,000
Holártica y Norte de Oriental, Este de Paleártica oriental	87,081,267
Bolivia	1,083,301
Oriental (Tailandia y Vietnam)	844,327
Paleártica (Paleártica occidental, y Este de Paleártica oriental) y Neotropical (Argentina)	30,330,851
Holártica, Costa Rica y Vietnam	77,382,312
Holártica (Paleártica y Neártica nororiental)	56,266,086
Semicosmopolita (Paleártica occidental, Mongolia, U.S.A., I. Seychelles, Hawái, Argentina, India: Kerala, y Antártica: I. Georgia del Sur)	36,178,664

Oriental (Sumatra y Filipinas)	771,651
Sumatra	473,481
Australia y Sumatra	8,155,781
Sumatra	473,481
Paleártica centromeridional	6,011,111
Java	1,267
Vietnam	331,212
Oriental y Japón	7,877,975
Sumatra	473,481
U.S.A. centrooriental	2,870,721
Oriental (Vietnam y Filipinas)	629,291
Paleártica y Vietnam	54,431,212
Oriental (Vietnam y Noreste de India)	593,442
Sumatra	473,481
Ecuador	283,561
Vietnam	331,212
Vietnam	331,212
Oriental (Vietnam y Camboya)	512,247
Oriental (Borneo y Sumatra)	1,225,417
Sumatra	473,481
Oriental (Borneo y Vietnam)	1,083,148
Oriental (Vietnam y Nepal)	478,728
Borneo	751,936
Semicosmopolita (Holártica, Norte de Oriental, Australia y Panamá)	88,827,873
Vietnam	331,212
Brasil	8,358,140
Neotropical (Ecuador y Perú)	1,563,557
Vietnam	331,212
Neártica septentrional	11,704,690
Oriental, Mediterránea oriental y Iran	9,726,692
Paleártica occidental	21,583,050
Paleártica, Oriental y Méjico	63,572,550
Holártica	77,000,000

Ecuador	283,561
Subtropical: Paleártica meridional (Mediterránea y Asia centrooccidental) y tropical: Senegal y Neotropical (I. Galápagos y Chile)	4,364,128
Mediterránea	2,085,292
Semicosmopolita (Paleártica, U.S.A. (Nuevo Méjico), Oriental: Bali, y Neotropical	73,420,695
Oriental y Australiana (Nuevas Hébridas)	7,512,190
U.S.A. oriental	2,276,528
Holártica, China suroriental y Panamá	78,912,525
Oriental (Tailandia y Vietnam)	844,327
Holártica (Europa, Paleártica oriental y U.S.A.: Virginia), India (Sikkim) e I. Seychelles.	18,151,669
Holártica e I. Seychelles	77,000,455
Boreoalpina	78,249,340
Vietnam	331,212
Holártica (Europa y Neártica septentrional)	21,884,690
Paleártica	54,100,000
Neotropical	19,000,000
Pantropical (excepto Australiana) y Paleártica meridional	58,933,333
Neotropical	19,000,000
Paleotropical y Paleártica meridional	47,633,333
Pantropical y Subtropical	81,935,554
Pantropical y subtropical	81,935,554
Semicosmopolita: Holártica, Oriental (Filipinas y China suroriental) y Norte de Neotropical	80,040,678
Mediterránea oriental	695,097
Cosmopolita (excepto Antártica)	134,940,000
Holártica, Noreste de Oriental y Nueva Zelanda.	81,334,693
Semicosmopolita: Holártica, Oriental, I. Seychelles y Méjico	86,473,005
Oriental (Java y Vietnam)	457,912
Oriental (Vietnam y Camboya)	512,247
Ecuador	283,561
Oriental	7,500,000
Paleártica, Etiópica (Península Arábiga y Etiopía), Méjico y Vietnam	60,641,262
Paleártica occidental (excepto Norte), Paleártica oriental (Este de Paleártica oriental e Irán) y Vietnam	19,896,140
Mediterránea occidental	1,390,194
Camerún	472,710

Tropical (Oriental, y Australiana: Islas del Pacífico) y Egipto	9,754,149
Mediterránea y Méjico	4,057,842
Oriental (Java y Vietnam)	457,912
Australia	7,682,300
Perú, Hawái, Malgache (I. Mauricio) y Japón	1,676,636
Pantropical, y Paleártica meridional.	45,637,644
Pantropical y Subtropical	81,935,554
Neotropical e India (Tripura)	19,010,492
Paleártica (Europa central e Irán)	2,522,084
Semicosmopolita (Holártica, India: Uttar Pradesh, Nueva Zelanda y Chile)	78,249,277
Holártica (Paleártica y Neártica septentrional)	65,804,690
Europa centromeridional	294,507
Etiópica, Polinesia (Tahití), Cuba y Vietnam	22,542,076
Paleártica: Paleártica occidental e Irán	23,114,644
Paleártica meridional (Mediterránea e Irán)	3,616,887
Europa centromeridional	294,507
Holártica (Paleártica occidental, Este de Paleártica oriental y U.S.A.)	37,420,835
Holártica: Paleártica y Neártica (U.S.A.: Virginia)	54,210,785
Paleártica y Nepal.	54,247,516
Mediterránea occidental	1,390,194
Holártica (Paleártica y U.S.A.: Carolina del Norte), Neotropical (Méjico y Brasil) y Oriental (Vietnam)	64,901,291
Mediterránea	2,085,292
Neotropical	19,000,000
Holártica (Paleártica occidental, Asia centooccidental y Neártica) y Oriental (Vietnam y Nepal)	42,746,545
Holártica (Paleártica y Canadá)	63,193,507
Holártica (Paleártica y Neártica septentrional), India (Sikkim) y Nueva Zelanda	66,076,323
Paleártica	54,100,000
Holártica y U.S.A. (Florida)	77,170,304
Paleártica occidental	21,583,050
Holártica (Paleártica y U.S.A.: Carolina del Norte) y China suroriental	56,076,497
Semicosmopolita: Australiana, Neotropical (América austral y Méjico) y Antártica (I. Príncipe Eduardo)	27,515,595
Neotropical (Argentina y Ecuador) y Antártica	17,020,206
Paleártica meridional	18,033,333

Paleártica occidental	21,583,050
Europa	10,180,000
Europa	10,180,000
Paleártica occidental (excepto Norte)	18,033,333
Este de Paleártica oriental	6,011,111
Paleártica meridional (Europa central y Paleártica oriental meridional)	7,001,600
Holártica y China suroriental	78,837,108
Semicosmopolita: Paleártica, Groenlandia, Oriental (Filipinas e India: Tripura) y Australia	62,090,962
Holártica y China suroriental	78,837,108
Paleártica (Europa occidental e Irán)	2,619,596
Mediterránea occidental	1,390,195
Vietnam	331,212
Ecuador	283,561
Pantropical (excepto Etiópica) y Paleártica meridional	54,713,333
Neotropical austral	5,067,041
Japón y Vietnam	709,187
Pantropical y subtropical	81,935,554
Neotropical	19,000,000
Norte de India	1,421,000
Oriental (Indonesia y Vietnam) y Australiana (Nuevas Hébridas [Vanuatu])	9,935,781
Polinesia (Tahití) y Oriental	7,501,044
Holártica y Neotropical	96,000,000
Holártica y Neotropical	96,000,000
Pantropical y subtropical	81,935,554
Mediterránea y Perú	3,365,288
Paleártica, Groenlandia y Australiana (Nueva Zelanda y Hawái)	56,547,348
Oriental	7,500,000
Java y Este de Paleártica oriental	6,137,811
Boreoalpina (Paleártica occidental, Paleártica oriental: excepto Este, y Neártica septentrional)	45,309,962
Java, China suroriental y Melanesia (I. Fiji)	1,982,081
Tropical (Neotropical, Etiópica: Ghana, y Oriental: Filipinas)	19,525,703
Oriental	7,500,000
Tropical (Etiópica: Malgache, Oriental: Java, y Australiana: Islas del Pacífico)	1,960,940

Paleártica meridional y Noreste de India	19,454,333
Paleártica	54,100,000
Semicosmopolita: Holártica, Etiópica (Sudáfrica) y Norte de Neotropical	79,126,437
Mediterránea oriental	695,097
Holártica (Paleártica occidental, Oeste de Siberia y U.S.A.)	33,909,724
Europa centromeridional	294,507
Holártica (Paleártica, y U.S.A.: Virginia)	54,210,785
Mediterránea occidental	1,390,194
Paleártica	54,100,000
Cosmopolita	148,940,000
Neotropical, Oriental (Filipinas y Vietnam) y Japón	20,007,357
Oriental y Este de Paleártica oriental	13,511,111
Neotropical	19,000,000
Semicosmopolita (Paleártica, Neártica oriental, Oriental, Australiana: Hawái, y Neotropical: Centroamérica)	69,771,834
Pantropical, Japón y "Polonia"	27,982,286
Mediterránea occidental	1,390,194
Mediterránea oriental	695,097
Mediterránea occidental	1,390,194
Paleártica centromeridional	6,011,111
Vietnam	331,212
Australiana (Indonesia: Lombok)	4,725
Japón y Vietnam	709,187
Paleártica, Etiopía y Neotropical	74,100,000
Holártica y Norte de Neotropical	77,905,400
Semicosmopolita (Boreoalpina, Australiana y Neotropical)	104,949,340
Neotropical	19,000,000
Paleártica occidental, Paleártica oriental (excepto Este), U.S.A. y Neotropical (Venezuela y Cuba)	46,915,211
Oriental (Vietnam y Camboya)	512,247
Cosmopolita (excepto Neotropical y Antártica)	115,940,000
Holárctica, Neotropical y China suroriental	97,837,108
Neotropical	19,000,000
Holártica e I. Santa Helena	77,000,123
Semicosmopolita (Holártica, Neotropical, Norte de Oriental, Nueva Zelanda, I. Santa Helena y Antártica)	114,334,816

Sureste de U.S.A., Vietnam y Hawái	1,852,192
Neotropical y Nueva Zelanda; Nueva Guinea	20,050,537
Neotropical (Bolivia y Perú)	2,363,297
Neotropical (Bolivia y Chile)	1,827,113
Oriental (Indonesia), Australiana (Micronesia: I. Marianas) y Japón	2,283,021
Oriental (Vietnam y Camboya)	512,247
Vietnam	331,212
Sumatra	473,481
Holártica	77,000,000
Holártica y Oriental	84,500,000
Borneo	751,936
Oriental (Vietnam y Nepal)	478,728
Vietnam	331,212
Vietnam	331,212
Vietnam	331,212
Neotropical	19,000,000
Paleártica occidental (excepto Norte), Mediterránea	14,107,514
Mediterránea occidental	1,390,194
Cosmopolita (excepto Antártica), Paleártica	134,940,000
Holártica y Norte de Neotropical	77,905,400
Holártica, Oriental (Himalaya) e I. Santa Helena., I. Azores	77,602,474
Holártica	77,000,000
Vietnam	331,212
Holártica, Nepal, Nueva Zelanda y Méjico	79,384,603
U.S.A. (Carolina del Norte)	126,161
Neotropical	19,000,000
Paleártica meridional	18,033,333
Neotropical (Paraguay y Brasil)	8,764,892
Neotropical (Paraguay y Pequeñas Antillas)	420,640
Java	1,267
U.S.A. (Nueva York)	1,413
Paleártica occidental	21,583,050

Holártica: Paleártica (Paleártica occidental) y Neártica (U.S.A.: Nueva York), y Neotropical: Antillas (La Española: R. Dominicana) y Ecuador	22,056,353
Holártica (Paleártica occidental, Asia centrooccidental y Neártica oriental)	30,551,219
Mediterránea	2,085,292
Cosmopolita	148,940,000
Holártica y Neotropical	96,000,000
Paleártica occidental, Este de Rusia asiática y Canadá	24,683,050
Holártica (Paleártica, Canadá y U.S.A)	73,020,182
Mediterránea oriental	695,097
Paleártica meridional (Europa centromeridional y Este de Rusia oriental)	3,394,507
Vietnam	331,212
Mediterránea	2,085,292
Oeste de Europa	1,088,001
Oriental	7,500,000
Cáucaso	400
Holártica (Paleártica y Neártica septentrional)	65,804,690
Holártica (Europa, Este de Rusia asiática y U.S.A.: Virginia)	17,243,385
Paleártica meridional y Norte de Oriental	22,103,489
Vietnam	331,212
Holártica (Paleártica y Neártica septentrional) y Etiopía & Paleártica meridional (Mediterránea y Asia centrooccidental)	66,804,690
Paleártica	54,100,000
Holártica e India (Sikkim)	77,007,096
Holártica: Paleártica y U.S.A. (Nuevo Méjico)	54,414,915
Paleártica y Norte de Oriental.	58,170,156
Boreal: Paleártica occidental, Noroeste de Siberia y Groenlandia	20,988,351
Oriental; Vietnam	7,500,000
Oriental	7,500,000
Tailandia	513,115
Oriental (Borneo y Vietnam)	1,083,148
Oriental (Vietnam y Nepal)	478,728
Oriental (Tailandia y Vietnam) y Japón	1,222,302
Etiópica y subantártica (I. San Pablo y Amsterdam), Ghana	22,327,594
Neotropical	19,000,000

Neotropical, India y Australiana (Nueva Zelanda y Australia)	30,234,306
Paleártica meridional (Mediterránea occidental e Irán)	2,921,790
Holártica y China suroriental, Japon	79,215,083
Holártica (Paleártica: Europa centromeridional, Mongolia y Noreste de China, y Neártica septentrional)	14,358,497
Holártica y Java	54,226,700
Tropical (Etiópica, Oriental y Polinesia) y subtropical (Cáucaso y Paleártica oriental meridional)	36,305,111
Vietnam	331,212
Paleártica meridional (Mediterránea e Irán) y Australia	11,299,187
Oriental	7,500,000
Borneo y Congo	3,096,794
Chile	743,812
Holártica e I. Santa Helena	54,100,123
Neotropical (Perú y Brasil)	9,638,136
Tropical (Neotropical y Oriental)	26,500,000
Neotropical y Oriental (Borneo)	19,751,936
Paleártica (Europa centromeridional e Irán)	1,826,102
Península Malaya	242,364
Semicosmopolita (Holártica, Etiópica y Neotropical)	118,100,000
Vietnam	331,212
Holártica, Oriental y Neotropical	103,500,000
Paleártica occidental (excepto Norte)	12,022,222
Holártica (Paleártica y U.S.A.: Nuevo Méjico) y Méjico	56,194,140
Mediterránea	2,085,292
Oriental	7,500,000
Polinesia y Oriental (Vietnam e India: Kerala)	664,075
Vietnam	331,212
Semicosmopolita (Holártica, Oriental: Vietnam y China suroriental, y Micronesia: I. Marianas)	13,568,834
Borneo	751,936
Vietnam	331,212
Java	1,267
Holártica (Paleártica occidental y Neártica septentrional)	33,287,740
Oriental	7,500,000
Islas del Pacífico y Sumatra	1,726,181

Vietnam	331,212
Vietnam	331,212
Oriental (Vietnam y Filipinas)	629,291
Holártica (Paleártica y U.S.A.) y Sudáfrica	65,147,712
Oriental (Filipinas y Sumatra)	771,651
Sumatra	473,481
Borneo	751,936
Vietnam	331,212
Nueva Guinea y Oriental (Indonesia)	2,680,569
Paleártica (Europa centromeridional y Suroeste de Siberia)	1,069,353
Neotropical	19,000,000
Vietnam	331,212
Vietnam	331,212
Holártica y Nepal	21,730,566
Europa	10,180,000
Neotropical	19,000,000
Holártica, I. Santa Helena, Nepal y Neotropical (Costa Rica y Bolivia)	78,282,000
Mediterránea oriental e Irán	2,226,692
Paleártica (Paleártica occidental y Corea) y Ceilán (Sri Lanka)	21,860,699
Holártica, Norte de Neotropical y Borneo	78,657,336
Holártica (Paleártica y U.S.A.: Míchigan)	54,350,494
Paleártica, Perú y Etiópica (I. Santa Helena e I. Reunión)	55,382,623
Holártica (Paleártica y Neártica) y Angola	78,246,700
Holártica y Uruguay	77,176,215
Pantropical (excepto Etiópica) y Subtropical	72,927,999
Mediterránea	2,085,292
Paleártica (Europa centromeridional y Suroeste de Siberia)	1,069,353
Cosmopolita (excepto Australiana y Antártica)	127,240,000
Holártica	10,180,000
Japón y Oriental	7,877,975
Paleártica meridional	18,033,333
Semicosmopolita (Holártica, Norte de Oriental, Australiana: Nueva Zelanda, Norte de Neotropical e I. Santa Helena)	82,240,216
Boreoalpina	78,249,340

Ecuador	283,561
Holártica (Paleártica oriental y Neártica septentrional) y Noreste de Oriental	32,837,293
Holártica	77,000,000
Holártica, China suroriental y Neotropical (Argentina e I. Galápagos)	81,581,808
Paleártica	54,100,000
Paleártica y Nepal	54,247,516
Oriental	7,500,000
Cosmopolita (excepto Antártica)	134,940,000
Vietnam	331,212
Vietnam	331,212
Semicosmopolita (Holártica, Etiópica: Senegal e I. Santa Helena, Oriental: Vietnam y China suroriental, y Hawái)	79,377,598
Oriental e Irán	9,031,595
Oriental, Paleártica oriental (Irán), Etiópica (Congo), Neotropical y Australiana (Nuevas Hébridas)	38,088,643
Argentina	2,736,690
Neotropical y Filipinas	19,298,170
Polinesia (Tahití), Etiópica (Congo) y Vietnam	2,677,114
Holártica, Oriental: China suroriental e India (Bengala Occidetal), Méjico y Hawái	80,915,035
Oriental (Java y Vietnam)	457,911
Vietnam	331,212
Semicosmopolita (Paleártica, Este de U.S.A., Oriental y Nueva Zelanda)	56,011,960
Europa y Mongolia	11,746,000
Pantropical y subtropical	81,935,554
Mediterránea	2,085,292
Holártica (Europa: excepto Norte, y U.S.A.: Carolina del Norte) y Java	9,271,785
Semicosmopolita: Holártica, China suroriental y Malgache (I. Mauricio)	78,839,148
Neotropical y Oriental	26,500,000
Semicosmopolita (Paleártica, U.S.A.: California, Malgache, Oriental y Hawái)	62,361,861
Tropical y subtropical: Paleártica (Mediterránea e Irán), U.S.A. (Nuevo Méjico), Neotropical (Costa Rica) y Oriental (Vietnam)	4,314,114
Europa	10,180,000
Paleártica occidental (excepto Norte), Paleártica oriental (Este de Paleártica oriental e Irán) y Vietnam	19,896,140
Paleártica y Venezuela	55,012,050
Mediterránea	2,085,292
Mediterránea	2,085,292

Holártica: excepto Este de Paleártica oriental y Senegal	71,181,419
Perú	1,279,996
Neotropical	19,000,000
Ceilán (Sri Lanka)	6,463
Pantropical y Subtropical	81,935,554
Oriental (Península Malaya y China suroriental)	2,079,472
Oriental (Borneo y Filipinas)	1,050,106
Semicosmopolita (Holártica y Pantropical: excepto Etiópica)	95,596,756
Islas del Pacífico	1,252,700
Mediterránea occidental	1,390,194
Vietnam	331,212
Ecuador	283,561
Ecuador	283,561
Europa	10,180,000
Paleártica meridional y Méjico	20,005,883
Neotropical e India	22,287,469
Neotropical	19,000,000
Cosmopolita (Paleártica meridional, Neártica y Pantropical)	89,533,333
Oriental (Java y Vietnam)	457,912
Semicosmopolita (Holártica, Paleotropical y Neotropical: Argentina y Bolivia)	116,041,441
Neotropical (Argentina e I. Galápagos)	2,744,700
Neotropical y Bután	19,038,394
Tropical: Neotropical (Perú y Brasil), Oriental (Vietnam e India: Orissa) y Etiópica (Congo y Etiopía)	13,469,913
Neotropical (Argentina e I. Galápagos)	2,745
Oriental	7,500,000
Cosmopolita (excepto Antártica)	134,940,000
Neotropical (Perú y Chile) y Vietnam	2,355,020
Semicosmopolita (excepto Antártica y Neotropical)	115,940,000
Semicosmopolita (Paleártica, India y Australia)	65,069,769
Semicosmopolita (Paleártica oriental meridional, China suroriental, Australia y Neotropical: Argentina y Panamá)	18,342,626
Neotropical	19,000,000
Europa	10,180,000
Holártica y Neotropical	96,000,000

Neártica y Japón	23,277,975
Borneo y Ecuador	1,035,497
Holártica	77,000,000
Holártica y Méjico	78,972,550
Península Malaya	242,364
Neotropical (Perú e I. Galápagos)	1,288,006
Méjico	1,972,550
Pantropical: Oriental (China suroriental y Vietnam), Australiana (Australia y Polinesia), Neotropical y Etiópica (Kenia y Etiopía), y subtropical	54,640,879
Perú	1,279,996
Paleártica (Europa, y Noroeste de Siberia)	10,917,700
Paleártica occidental	18,033,333
Paleártica: Paleártica occidental (excepto Norte) e Irán	13,553,817
Paleártica e India (Cachemira)	54,322,000
Europa centrooccidental	247,662
Holártica (Paleártica occidental, Irán y U.S.A.) e I. Santa Helena	32,941,443
Mediterránea oriental y Paleártica oriental meridional	6,706,208
Europa, Irán e India (Megalaya), Mediterránea oriental	12,429,121
Neotropical (Brasil y Ecuador)	8,641,701
Neotropical (Bolivia y Ecuador)	1,366,862
Argentina	2,736,690
Ecuador	283,561
Australiana (Polinesia: Tahití), Oriental (Vietnam e India: Bengala Occidental) y Neotropical (I. Galápagos)	429,018
Borneo	751,936
Pantropical (excepto Australiana) y Japón	41,277,975
Paleártica (Europa y Paleártica oriental meridional)	16,191,111
Paleártica meridional (Europa centromeridional e Irán)	1,826,102
Paleártica occidental (excepto Norte)	12,022,222
Holártica (Europa centrooccidental y Canadá)	9,341,129
Holártica (Paleártica, y Canadá)	63,193,507
Holártica (Paleártica occidental, Este de Rusia asiática y Neártica).	31,655,933
[Holártica y Argentina, Holártica (Paleártica y Canadá), Mediterránea, Paleártica, Holártica (Paleártica occidental, Este de Rusia asiática y Neártica)] = Holarctic + Argentina	79,736,690

Europa centromeridional e India (Tripura)	304,999
Neotropical, Oriental y Holártica meridional (Paleártica meridional y U.S.A.: Luisiana)	44,667,597
Paleárctica; Europa centromeridional	54,100,000
Neártica (U.S.A.: Carolina del Norte, y Canadá)	9,232,896
India (Kerala)	38,863
Holártica (U.S.A.: Carolina del Norte, y Este de Rusia asiática)	7,091,989
Paleártica (Europa y Oeste de Siberia) y Vietnam	12,226,196
Semicosmopolita: Holártica (Neártica oriental, Europa y Este de Rusia asiática), El Salvador, Nueva Zelanda y Vietnam	25,382,723
Holártica	77,000,000
Oriental (Indonesia y Vietnam)	2,235,781
Neotropical	19,000,000
Vietnam	331,212
Malgache (I. Mauricio)	204
Java	1,267
Bali	578
Nueva Guinea, Oriental y Brasil	16,644,140
Neotropical (Perú y Centroamérica)	1,801,872
Paleártica (Europa y Este de Rusia asiática)	17,132,600
Semicosmopolita: Holártica, Oriental y Nueva Zelanda	84,764,537
Pantropical y Japón	48,977,975
Nueva Guinea	786,000
Tropical (Neotropical: Perú y Panamá, Australiana, Etiopía e India: Bengala Occidental) y Subtropical	9,144,165
Oriental (Indonesia y Vietnam)	2,235,781
Perú y Brasil	9,638,136
Este de Paleártica oriental y China suroriental	7,848,219
Java	1,267
Borneo	751,936
Borneo	751,936
Oriental (Sumatra e India: Arunachal Pradesh)	557,224
Holártica	77,000,000
Vietnam y Méjico	2,303,762
Tropical (Oriental y Neotropical) y Paleártica meridional	44,533,333
Oriental (Filipinas y Vietnam)	626,382

Oriental (Borneo y Vietnam)	1,083,148
Paleártica	54,100,000
Semicosmopolita: Paleártica (Paleártica occidental, Irán y Japón), U.S.A. (Virginia), Oriental, I. Galápagos y Nueva Zelanda	31,375,952
Cosmopolita	148,940,000
Cosmopolita	148,940,000
Mediterránea, Oriental: Filipinas y Ceilán [Sri Lanka], y Melanesia (I. Fiji)	2,466,366
Pantropical y subtropical (Paleártica meridional)	6,663,333
Neotropical	19,000,000
Paleártica meridional	18,033,333
Neotropical	19,000,000
Oriental (Indonesia) y Nueva Guinea	2,690,569
Semicosmopolita (Holártica, Etiópica: I. Santa Helena, Australiana, Neotropical y Oriental: Vietnam)	104,031,335
U.S.A	9,826,675
Cosmopolita: Holártica, Oriental (China suroriental y Noreste de India), Etiopía y Neotropical	99,099,338
Semicosmopolita: Paleártica meridional (Cáucaso y Sureste de Paleártica oriental), Oriental, Etiópica (Zambia) y Netropical (Ecuador)	26,569,512
Cáucaso	400
Perú	1,279,996
Holártica: Paleártica y Canadá	63,193,507
Holártica (Paleártica y U.S.A.: Virginia)	54,100,000
Paleártica occidental (menos fecuente en el Norte)	21,583,049
Borneo	751,936
Ruanda	26,338
Semicosmopolita: Holártica, Neotropical, I. Santa Helena y Australiana (Nueva Zelanda e Islas del Pacífico)	103,700,123
Paleártica (Europa meridional y Este de Rusia asiática)	4,417,044
Vietnam	331,212
Oriental y Este de Paleártica oriental	13,511,111
Vietnam	331,212
Pantropical y subtropica	81,935,554
África centrooriental	4,350,015
Holártica (Paleártica occidental, Paleártica oriental meridional, y U.S.A.: Míchigan) y Méjico	26,267,488
Paleártica y China suroriental	55,937,108
Colombia	1,038,700

Neotropical, U.S.A. meridional y Oriental (Borneo y Vietnam)	22,191,555
Vietnam	331,212
Oriental y Este de Paleártica oriental	13,511,111
Holártica: Paleártica occidental, Paleártica oriental (I. Kuriles) y Neártica septentrional	33,298,244
Paleártica occidental, Este de Rusia asiática y Neotropical (Argentina y Perú)	29,002,619
Europa meridional	1,317,044
Nueva Guinea	786
Oriental y Paleártica: Crimea y Corea	7,739,863

s. Chapman and Hall, New York, USA, pp 8-99

3–373
