# Supplementary material for: “Jack‐of‐all‐trades” is parthenogenetic
Source: Ecol Evol. 2022 Jun 23;12(6):e9036. doi: 10.1002/ece3.9036 (PMC9219104; doi:10.1002/ece3.9036)
Supplement: Supplementary file 3 — Appendix S3 [file ECE3-12-e9036-s003.pdf]

Appendix, Table 3: Oribatid mite species, their taxonomic affiliation, reproductive mode and geographic range (km²), their NCBI accession numbers and length of the 18S fragment used for the molecular phylogeny in this study. NCBI data were used to construct a solid family level molecular phylogeny based on all taxa listed in Appendix Table 2 to test for phylogenetic independence of geographic range among ori Taxa kept for phylogenetic trait analysis (one species per monophyletic clade) are indicated by asterisks (*). Species from NCBI may deviate from taxa used for our study, but represent the same genus or family.

**Oribatid mite species**	**Oribatid mite species downloaded from NCBI, deviating from Table 2**	**Taxon**
Aphelacarus acarinus (Berlese, 1910)		Palaeosomata
Ctenacarus aranaeola (Grandjean, 1932)*		Palaeosomata
Palaeacarus hystricinus Trägardh, 1932*		Palaeosomata
Brachychthonius berlesei Willmann, 1928*	*Brachychthonius bimaculatus* *	Enarthronota
Liochthonius alpestris (Forsslund, 1958)	*Liochthonius* sp.	Enarthronota
Liochthonius neglectus Moritz, 1976		Enarthronota
Liochthonius peduncularius (Strenzke, 1951)		Enarthronota
Sellnickochthonius cricoides (Weis-Fogh, 1948)		Enarthronota
Sellnickochthonius zelawaiensis (Sellnick, 1928)		Enarthronota
Cosmochthonius lanatus (Michael, 1885)*		Enarthronota
Eniochthonius minutissimus (Berlese, 1903)*		Enarthronota
Haplochthonius sanctaeluciae Bernini, 1973*	*Haplochthonius simplex**	Enarthronota
Eohypochthonius salicifolius Hammer, 1979*	*Eohypochthonius gracilis**	Enarthronota
Hypochthonius luteus Oudemans, 1917		Enarthronota
Hypochthonius rufulus C.L. Koch, 1835		Enarthronota
Malacoangelia remigera Berlese, 1913		Enarthronota
Lohmannia hungarorum Mahunka, 1980*	*Lohmannia banksi* *	Enarthronota
Apoplophora cristata Mahunka, 1991*	*Apoplophora* sp. *	Enarthronota
Archoplophora rostralis (Willmann, 1930)		Enarthronota
Mesoplophora leviseta Hammer, 1979	*Mesoplophora abscondita*	Enarthronota
Mesoplophora michaeliana Berlese, 1904*	*Mesoplophora cubana**	Enarthronota
Pterochthonius angelus (Berlese, 1910)*		Enarthronota
Sphaerochthonius splendidus (Berlese, 1904)*	*Sphaerochthonius* sp.*	Enarthronota
Gozmanyina majestus (Marshall & Reeves, 1971)*		Enarthronota

Gehypochthonius rhadamanthus Jacot, 1936*	*Gehypochthonius urticinus* *	Parhyposomata
Epilohmannia minuta Berlese, 1920*	*Epilohmannia* sp.*	Mixonomata
Eulohmannia ribagai (Berlese, 1910)*		Mixonomata
Acrotritia duplicata (Grandjean, 1953)*	*Rhysotritia duplicata* *	Mixonomata
Nehypochthonius porosus Norton & Metz, 1980*		Mixonomata
Oribotritia berlesei (Michael, 1898)*	*Indotritia krakatauensis**	Mixonomata
Atropacarus striculus (Koch, 1835)		Mixonomata
Phthiracarus bryobius Jacot, 1930*	*Phthiracarus* sp.*	Mixonomata
Steganacarus applicatus (Sellnick, 1920)		Mixonomata
Steganacarus magnus (Nicolet, 1855)		Mixonomata
Camisia biurus (C.L. Koch, 1839)		Desmonomata
Camisia horrida (Hermann, 1804)		Desmonomata
Camisia invenusta (Michael, 1888)		Desmonomata
Camisia spinifer (C.L. Koch, 1835)		Desmonomata
Heminothrus targionii (Berlese, 1885)	*Heminothrus paolianus*	Desmonomata
Platynothrus peltifer (C.L. Koch, 1839)*		Desmonomata
Hermannia gibba (C.L. Koch, 1839)*		Desmonomata
Malaconothrus gracilis Hammen, 1952		Desmonomata
Malaconothrus monodactylus (Michael, 1888)*		Desmonomata
Cyrthermannia florens Balogh & Mahunka, 1980		Desmonomata
Nanhermannia coronata Berlese, 1913*		Desmonomata
Nanhermannia elegantissima Hammer, 1958		Desmonomata
Nanhermannia nana (Nicolet, 1855)		Desmonomata
Nothrus borussicus Sellnick, 1928		Desmonomata
Nothrus silvestris Nicolet, 1855		Desmonomata
Nothrus silvicus Jacot, 1937		Desmonomata
Nothrus willmanni Mahunka, 1983*		Desmonomata
Afronothrus incisivus Wallwork, 1961		Desmonomata
Allonothrus russeolus Wallwork, 1960		Desmonomata
Archegozetes longisetosus Aoki, 1965		Desmonomata

Mucronothrus nasalis (Willmann, 1929)		Desmonomata
Trhypochthoniellus longisetosus (Berlese, 1904)*	*Trhypochthoniellus crassus**	Desmonomata
Trhypochthonius americanus (Ewing, 1908)		Desmonomata
Trhypochthonius tectorum (Berlese, 1896)		Desmonomata
Achipteria coleoptrata (Linné, 1758)*		Brachypylina
Tecteremaeus incompletus Mahunka, 1988*		Brachypylina
Caleremaeus monilipes (Michael, 1882)*		Brachypylina
Epieremulus granulatus (Balogh & Mahunka, 1979)*		Brachypylina
Carabodes coriaceus C.L. Koch, 1835		Brachypylina
Carabodes labyrinthicus (Michael, 1879)*		Brachypylina
Carabodes subarcticus Trägårdh, 1902		Brachypylina
Odontocepheus elongatus (Michael, 1879)*		Brachypylina
Cepheus dentatus (Michael, 1888)		Brachypylina
Cepheus latus C.L. Koch, 1835*		Brachypylina
Ceratoppia bipilis (Hermann, 1804)*		Brachypylina
Edwardzetes edwardsi (Nicolet, 1855)*		Brachypylina
Euzetes globulus (Nicolet, 1855)*		Brachypylina
Oromurcia bicuspidata Thor, 1930*	*Oromurcia sudetica* *	Brachypylina
Trichoribates incisellus (Kramer, 1897)	*Trichoribates trimaculatus*	Brachypylina
Chamobates pusillus (Berlese, 1895)		Brachypylina
Chamobates subglobulus (Oudemans, 1900)*		Brachypylina
Chamobates voigtsii (Oudemans, 1902)		Brachypylina
Cymbaeremaeus cymba (Nicolet, 1855)*		Brachypylina
Scapheremaeus bicornutus Hammer, 1971*	*Scapheremaeus nakanoshimensis**	Brachypylina
Scapheremaeus foveolatus Mahunka, 1987	*Scapheremaeus palustris*	Brachypylina
Damaeus clavipes (Hermann, 1804)*		Brachypylina
Damaeus flagelloides (Norton, 1979)		Brachypylina
Dameobelba minutissima (Sellnick, 1920)		Brachypylina
Metabelba papillipes (Nicolet, 1855)		Brachypylina
Beckiella capitulum Balogh & Mahunka, 1978*		Brachypylina
Eueremaeus oblongus (Koch, 1835)*		Brachypylina
Eremaeozetes lineatus Mahunka, 1985*	*Eremaeozetes* sp.*	Brachypylina

Acrogalumna longipluma (Berlese, 1904)		Brachypylina
Galumna lanceata (Oudemans, 1900)*		Brachypylina
Pilogalumna crassiclava (Berlese, 1914)	*Pilogalumna rosauraruizae*	Brachypylina
Gymnodamaeus bicostatus (C.L. Koch, 1835)*		Brachypylina
Peloribates europaeus Willmann, 1953*	*Peloribates acutus* *	Brachypylina
Rostrozetes ovulum (Berlese, 1908)*		Brachypylina
Rostrozetes shibai Aoki, 1976*	*Rostrozetes nebulosus* *	Brachypylina
Hemileius perforatoides (Hammer, 1979)	*Hemileius singularis*	Brachypylina
Ampullobates ecuadoriensis Ermilov et al., 2013*		Brachypylina
Hermannobates monstruosus Hammer, 1961		Brachypylina
Adoristes ovatus (C.L. Koch, 1839)		Brachypylina
Dorycranosus acutus (Pschorn-Walcher, 1951)*	*Liacarus coracinus**	Brachypylina
Liebstadia humerata Sellnick, 1928*		Brachypylina
Microtegeus similis Balogh & Mahunka, 1980*		Brachypylina
Unguizetes cattienensis Ermilov & Anichkin, 2011*	*Unguizetes incertus* *	Brachypylina
Poroliodes farinosus (C.L. Koch, 1840)*		Brachypylina
Berniniella sigma (Strenzke, 1951)	*Oppiella splendens*	Brachypylina
Dissorhina ornata (Oudemans, 1900)		Brachypylina
Gittella variabilis Ermilov et al., 2013*		Brachypylina
Oppia nitens C.L. Koch, 1836	*Rhinoppia nasuta*	Brachypylina
Oppiella nova (Oudemans, 1902)*		Brachypylina
Joelia fiorii (Coggi, 1898)*	*Paralamellobates misella**	Brachypylina
Oribatula interrupta Willmann, 1939	*Oribatula sakamorii*	Brachypylina
Oribatula tibialis (Nicolet, 1855)		Brachypylina
Phauloppia lucorum (C.L. Koch, 1841)*		Brachypylina
Oribella pectinata (Michael, 1885)*	*Pantelozetes paolii**	Brachypylina
Eupelops acromios (Hermann, 1804)		Brachypylina
Eupelops plicatus (C.L. Koch, 1835)*		Brachypylina
Pheroliodes intermedius (Hammer, 1961)*	*Pheroliodes* sp.*	Brachypylina
Solenozetes carinatus (Hammer, 1961)*		Brachypylina
Protoribates capucinus Berlese, 1908*	*Protoribates hakonensis**	Brachypylina
Punctoribates punctum (C.L. Koch, 1839)*		Brachypylina
Rhynchoribates mirus Beck, 1961*		Brachypylina

Scheloribates ascendens Weigmann & Wunderle, 1990		Brachypylina
Scheloribates initialis (Berlese, 1908)*	*Hemileius microclava**	Brachypylina
Scheloribates pallidulus (C.L. Koch, 1841)*		Brachypylina
Scutovertex sculptus Michael, 1879*		Brachypylina
Tectocepheus minor Berlese, 1903*		Brachypylina
Tectocepheus velatus Michael, 1880		Brachypylina
Dolicheremaeus dorni (Balogh, 1937)*		Brachypylina
Plenotocepheus neotropicus Ermilov et al., 2013*		Brachypylina
Xenillus clypeator Robineau-Desvoidy, 1839*	*Xenillus discrepans* *	Brachypylina
Xenillus tegeocranus (Hermann, 1804)		Brachypylina
Zetorchestes novaguineanus Krisper, 1987	*Zetorchestes micronychus*	Brachypylina
**outgroups**		
Demodex brevis		Cheyletoidea
Balaustium sp.		Erythraeoidea
Ameroseius plumosus		Gamasina
Polyaspis sp.		Uropodina
Sternothyrus braueri		Holothyrida
Opilioacarus texanus		Opilioacarida
Opilioacarid SJD-2001		Opilioacarida
Neocarus bajacalifornicus chamelaensis		Opilioacarida

batid mite species.

Family	reproductive mode	area (km^2)	NCBI no.	18S (bp)
Aphelacaridae	sexual	118,336,000	DQ648879	505
Ctenacaridae	sexual	81,935,554	EU433991	1937
Palaeacaridae	parthenogenetic	79,215,083	EF204472	1704
Brachychthoniidae	parthenogenetic	77,000,000	OL839311	1945
Brachychthoniidae	parthenogenetic	54,100,000	JQ000035	1859
Brachychthoniidae	parthenogenetic	21,583,050	OL839308	1931
Brachychthoniidae	parthenogenetic	10,180,000	OL839310	1901
Brachychthoniidae	parthenogenetic	65,069,769	OL839309	1945
Brachychthoniidae	parthenogenetic	77,000,000	MH198174	1725
Cosmochthoniidae	sexual	125,600,000	JN585919	2132
Eniochthoniidae	parthenogenetic	134,940,000	KR081609	1842
Haplochthoniidae	parthenogenetic	4,364,128	KY922210	2158
Hypochthoniidae	parthenogenetic	878,636	EF203777	1850
Hypochthoniidae	parthenogenetic	81,334,693	EU152475	1707
Hypochthoniidae	parthenogenetic	86,473,005	KR081618	1813
Hypochthoniidae	parthenogenetic	81,935,554	JN585911	1651
Lohmanniidae	parthenogenetic	1,390,195	AF022036	1759
Mesoplophoridae	sexual	242,364	JN585917	1779
Mesoplophoridae	parthenogenetic	37,914,527	JN585918	1826
Mesoplophoridae	sexual	1,960,940	JN585912	1,797
Mesoplophoridae	sexual	19,454,333	EU432217	1835
Pterochthoniidae	parthenogenetic	80,915,035	EU432214	1713
Sphaerochthoniidae	sexual	54,640,879	JN585916	2142
Trichthoniidae	parthenogenetic	11,704,690	EU433993	1800

Gehypochthoniidae	parthenogenetic	88,827,873	EU433994	1825
Epilohmanniidae	parthenogenetic	32,290,167	EU432213	1755
Eulohmanniidae	parthenogenetic	78,837,108	EU432211	1635
Euphthiracaridae	parthenogenetic	54,478,728	EF091417	1797
Nehypochthoniidae	parthenogenetic	1,852,192	EF081308	1741
Oribotritiidae	sexual	58,170,156	JN585920	1625
Phthiracaridae	parthenogenetic	92,721,876	EF091416	1742
Phthiracaridae	sexual	78,282,000	KR081629	1824
Phthiracaridae	sexual	1,390,195	GQ864301	1672
Phthiracaridae	sexual	32,941,443	AF022040	1736
Camisiidae	parthenogenetic	78,837,108	EF081302	1741
Camisiidae	parthenogenetic	82,975,556	EU432207	1624
Camisiidae	parthenogenetic	82,975,556	EU432208	1624
Camisiidae	parthenogenetic	89,503,713	EF091420	1741
Camisiidae	parthenogenetic	78,912,525	EF091423	1741
Camisiidae	parthenogenetic	82,240,216	EF091422	1795
Hermanniidae	sexual	77,000,455	EF091426	1739
Malaconothridae	parthenogenetic	96,000,000	JQ000044	1753
Malaconothridae	parthenogenetic	96,000,000	KR081621	1813
Nanhermanniidae	parthenogenetic	19,000,000	KR081606	1823
Nanhermanniidae	parthenogenetic	97,837,108	EF091421	1741
Nanhermanniidae	parthenogenetic	19,000,000	KR081623	1717
Nanhermanniidae	parthenogenetic	114,334,816	OL839307	1767
Nothridae	parthenogenetic	77,905,400	KY922216	1776
Nothridae	parthenogenetic	79,384,603	EF091425	1766
Nothridae	parthenogenetic	126,161	EF204473	1700
Nothridae	parthenogenetic	19,000,000	KR081625	1808
Trhypochthoniidae	parthenogenetic	27,604,311	JQ000045	1753
Trhypochthoniidae	parthenogenetic	22,477,975	AF022025	1812
Trhypochthoniidae	parthenogenetic	26,500,000	HQ661379	1700

Trhypochthoniidae	parthenogenetic	104,949,340	EF081299	1741
Trhypochthoniidae	parthenogenetic	104,031,335	EF081300	1741
Trhypochthoniidae	parthenogenetic	9,826,675	JQ000046	1776
Trhypochthoniidae	parthenogenetic	99,099,338	AF022041	1737
Achipteriidae	sexual	65,811,786	EF091418	1771
Arceremaeidae	sexual	1,083,148	KR081635	1820
Caleremaeidae	sexual	23,555,599	OL839305	1781
Caleremaeidae	sexual	1,188,961	KR081610	1809
Carabodidae	sexual	21,693,835	EF093787	1799
Carabodidae	sexual	78,972,550	KX397629	1822
Carabodidae	sexual	28,213,333	EF091429	1789
Carabodidae	sexual	141,300	KY922219	1777
Cepheidae	sexual	13,280,000	OL839306	1795
Cepheidae	sexual	78,984,623	EU432206	1624
Ceratoppiidae	sexual	81,592,031	EU432204	1624
Ceratozetidae	sexual	2,951,293	MH198178	1827
Ceratozetidae	sexual	30,330,851	AF022030	1815
Ceratozetidae	sexual	24,538,068	EU432194	1625
Ceratozetidae	sexual	63,193,507	EU432195	1625
Chamobatidae	sexual	54,100,000	EU432188	1624
Chamobatidae	sexual	26,517,528	EU432190	1624
Chamobatidae	sexual	30,330,851	EU432189	1624
Cymbaeremaeidae	sexual	54,107,096	EU432201	1624
Cymbaeremaeidae	sexual	1,252,700	LC367334	1797
Cymbaeremaeidae	sexual	331,212	EU433989	1823
Damaeidae	sexual	21,583,049	KR081607	1824
Damaeidae	sexual	1,027,373	KR081608	1792
Damaeidae	parthenogenetic	24,683,049	MH198179	1810
Damaeidae	sexual	33,909,724	MH198180	1825
Dampfiellidae	sexual	109,820	KR081602	1719
Eremaeidae	sexual	78,837,107	GQ864287	1671
Eremaeozetidae	sexual	97,000,000	EU432187	1639

Galumnidae	sexual	101,240,508	GQ864304	1664
Galumnidae	sexual	54,431,212	KX397630	1809
Galumnidae	sexual	1,069,353	KJ423065	686
Gymnodamaeidae	sexual	77,000,000	KR081614	1802
Haplozetidae	sexual	56,194,140	AB818529	1824
Haplozetidae	parthenogenetic	81,935,554	HM070342	1734
Haplozetidae	sexual	2,079,472	KR081633	1795
Hemileiidae	sexual	7,512,190	AB818531	1806
Hermanniellidae	sexual	283,561	KR081601	1824
Hermanniellidae	sexual	19,000,000	KR081617	1824
Liacaridae	sexual	24,308,220	GQ864286	1671
Liacaridae	sexual	23,114,644	KR081619	1824
Liebstadiidae	sexual	42,746,545	KR081620	1803
Microtegeidae	sexual	19,000,000	KR081622	1807
Mochlozetidae	sexual	331,212	KR081636	1826
Neoliodidae	sexual	54,247,516	EF203779	1515
Oppiidae	sexual	294,507	OL839303	1753
Oppiidae	sexual	56,030,512	MH198181	1823
Oppiidae	sexual	1,563,557	KR081612	1820
Oppiidae	sexual	30,551,219	OL839304	1816
Oppiidae	parthenogenetic	148,940,000	KR081626	1751
Oribatellidae	sexual	4,057,842	KT781157	1593
Oribatulidae	sexual	66,804,690	AB818530	1837
Oribatulidae	sexual	77,007,096	EU433990	1819
Oribatulidae	sexual	21,730,566	EU432198	1648
Oribellidae	sexual	54,414,915	MH198177	741
Phenopelopidae	sexual	63,121,316	EU432192	1624
Phenopelopidae	sexual	55,928,639	EF091419	1807
Pheroliodidae	sexual	19,000,000	KR081628	1732
Plasmobatidae	sexual	1,288,006	KR081634	1791
Protoribatidae	parthenogenetic	134,940,000	AB818528	1798
Punctoribatidae	sexual	56,011,960	MH198175	1606
Rhynchoribatidae	sexual	1,279,996	KR081632	1824

Scheloribatidae	sexual	10,180,000	EU432199	1627
Scheloribatidae	sexual	73,227,370	KR081616	1801
Scheloribatidae	sexual	134,940,000	AB818527	1800
Scutoverticidae	sexual	115,940,000	GQ864305	1679
Tectocepheidae	parthenogenetic	31,375,952	EF093778	1805
Tectocepheidae	parthenogenetic	148,940,000	EF093781	1805
Tetracondylidae	sexual	294,597	MG719344	1717
Tetracondylidae	sexual	283,561	KR081631	1797
Xenillidae	sexual	26,267,488	EU432203	1624
Xenillidae	sexual	55,937,108	KR081637	1826
Zetorchestidae	sexual	786,000	MK014975	1744
Demodicidae	sexual		HQ727999	1822
Erythraeidae	sexual		LC260406	1794
Ascoidea	sexual		MG547400	1473
Polyaspididae	sexual		KY922099	1756
Holothyridae	sexual		AY620912	1774
Opilioacaridae	sexual		AF115375	1784
Opilioacaridae	sexual		AF287235	1747
Opilioacaridae	sexual		MF281419	1706
