# Supplementary figures and images for: “Jack‐of‐all‐trades” is parthenogenetic
Source: Ecol Evol. 2022 Jun 23;12(6):e9036. doi: 10.1002/ece3.9036 (PMC9219104; doi:10.1002/ece3.9036)

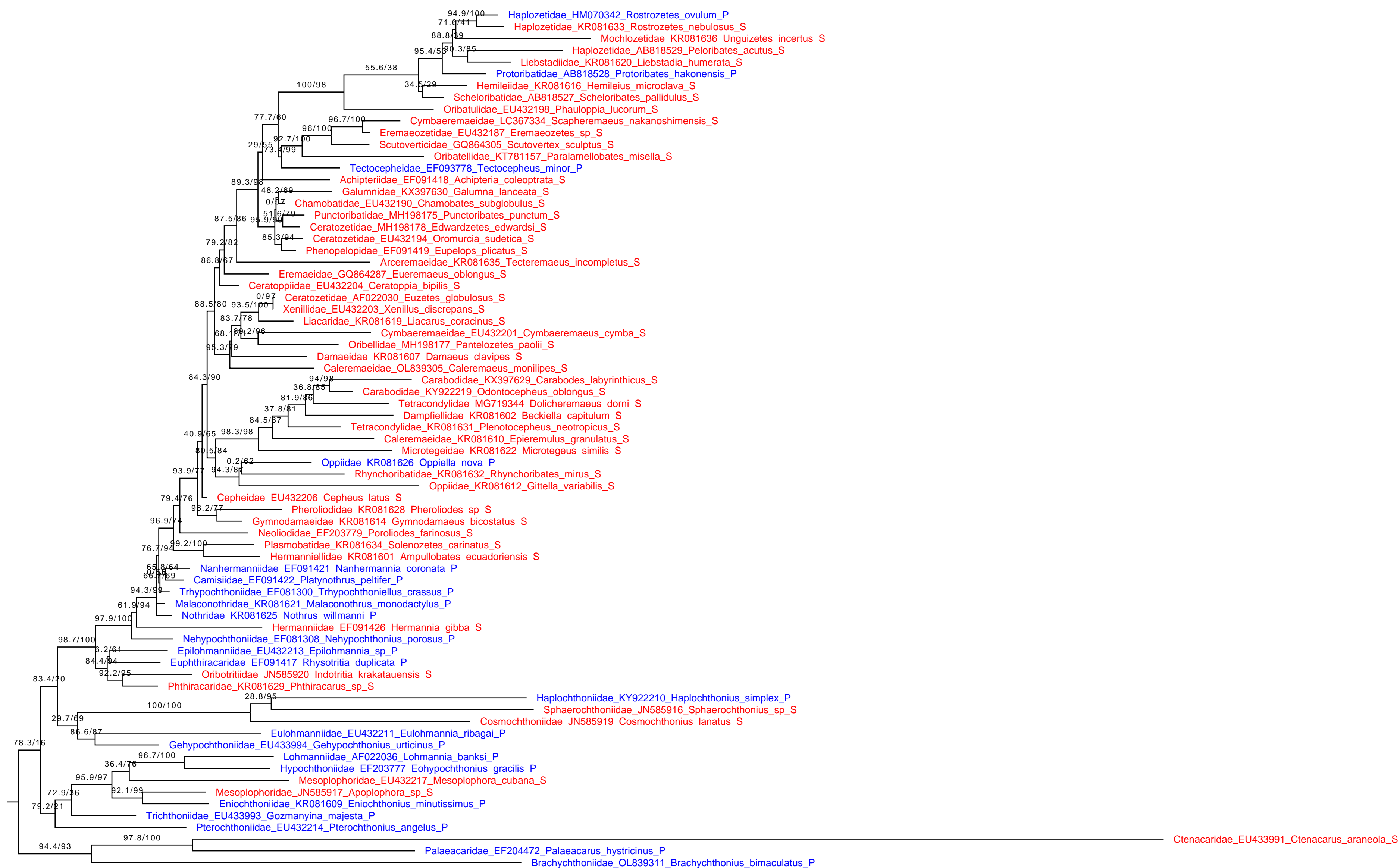

Supplement: Supplementary file 4 — Appendix S4 [file ECE3-12-e9036-s004.pdf]
